# Structural insight into how the human helicase subunit MCM2 may act as a histone chaperone together with ASF1 at the replication fork

**DOI:** 10.1093/nar/gkv021

**Published:** 2015-01-23

**Authors:** Nicolas Richet, Danni Liu, Pierre Legrand, Christophe Velours, Armelle Corpet, Albane Gaubert, May Bakail, Gwenaelle Moal-Raisin, Raphael Guerois, Christel Compper, Arthur Besle, Berengère Guichard, Genevieve Almouzni, Françoise Ochsenbein

**Affiliations:** 1 CEA, iBiTec-S, SB2SM, Laboratoire de Biologie Structurale et Radiobiologie, France; 2 Institute for Integrative Biology of the Cell (I2BC), CEA, CNRS, Université Paris-Sud, Batiment 144, Gif-sur-Yvette, F-91191, France; 3 Synchrotron SOLEIL, F-91190, Gif-sur-Yvette, France; 4 Laboratoire d'Enzymologie et de Biologie Structurale, CNRS UPR 3082, 1 avenue de la Terrasse, Gif-sur-Yvette, F-91190, France; 5 Institut Curie, Centre de Recherche, CNRS UMR 3664, Equipe Labellisée Ligue contre le Cancer, and Université Pierre et Marie Curie, Université Sorbonne PSL*, Paris, F-75248, France

## Abstract

MCM2 is a subunit of the replicative helicase machinery shown to interact with histones H3 and H4 during the replication process through its N-terminal domain. During replication, this interaction has been proposed to assist disassembly and assembly of nucleosomes on DNA. However, how this interaction participates in crosstalk with histone chaperones at the replication fork remains to be elucidated. Here, we solved the crystal structure of the ternary complex between the histone-binding domain of Mcm2 and the histones H3-H4 at 2.9 Å resolution. Histones H3 and H4 assemble as a tetramer in the crystal structure, but MCM2 interacts only with a single molecule of H3-H4. The latter interaction exploits binding surfaces that contact either DNA or H2B when H3-H4 dimers are incorporated in the nucleosome core particle. Upon binding of the ternary complex with the histone chaperone ASF1, the histone tetramer dissociates and both MCM2 and ASF1 interact simultaneously with the histones forming a 1:1:1:1 heteromeric complex. Thermodynamic analysis of the quaternary complex together with structural modeling support that ASF1 and MCM2 could form a chaperoning module for histones H3 and H4 protecting them from promiscuous interactions. This suggests an additional function for MCM2 outside its helicase function as a proper histone chaperone connected to the replication pathway.

## INTRODUCTION

The nucleosome is the universal repeating unit of chromatin. Its core particle contains 147 base pair of DNA wrapped around an octamer of two copies of the four core histones H2A, H2B, H3 and H4 (1). This particle exists in various forms using distinct histone variants for H3 (in humans, the S phase subtypes H3.1 and H3.2, the H3.3 variant deposited at all phases of the cell cycle and the centromeric CENP-A (2–4)), and for H2A (macro H2A, H2AX, H2AZ…(4)) as well as the large repertoire of post-transcriptional modifications (PTMs), also called the ‘epigenetic code’ (5). Together these combinations can establish a chromatin landscape regulating gene expression programs. Mechanisms that regulate chromatin states during cell cycle are thus essential for maintaining the cellular identity upon cell division. Consistent with this central function, deregulation of histone marks promotes tumoral progression (6). During S phase, DNA replication is accompanied by histone eviction and re-association downstream the replication fork. This raises the question of how parental histones and their marks (PTM and variants) are handled at the fork and whether they re-associate at the same position after fork passage. Doubling of the DNA is associated with doubling of histones through simultaneous incorporation of newly synthesized histones. Newly synthesized histones differ from parental histones in their specific PTMs, modifications that can be removed during maturation so as to place modifications that compares to parental chromatin in a second step. In the nucleosome, each histone is present in two copies. Thus, a mechanism that combines parental and newly synthesized histones in one nucleosome in a semi-conservative fashion has been considered as an attractive hypothesis. To date, however, existing studies (7), based on the *in vivo* stability of the histone H3-H4 hetero-tetramers that carry most of the epigenetic marks, favor a model in which the template to copy histone marks would use a neighboring nucleosome. This view has been challenged when realizing that (i) histones H3-H4 can be incorporated as dimers *in vivo* (8) and (ii) one histone chaperone dedicated to H3-H4 processing, ASF1 (Anti-Silencing Function 1) binds the histone dimer in a competitive manner with tetramer formation and dissociates the tetramer *in vitro* (9–12). Splitting the H3-H4 tetramer would thus allow the mixing of parental and newly synthetized histone dimers, but this does not rule out re-association to reconstitute the pre-existing tetramers. Finally, recent experiments revisiting this question by SILAC approaches showed that histone rarely mix, especially for the S phase-specific variant H3.1 (13). It has thus been proposed that parental and newly synthesized histones may exploit distinct pathways during DNA replication (14,15). However, only a partial knowledge of the actors in these pathways is currently available and their precise roles remain to be discovered.

Histone chaperones escort histones throughout their cellular life and are key factors in handling histones during replication (16,17). Among them, the CAF-1 complex (chromatin assembly factor 1) is key as a histone H3-H4 deposition factor coupled to DNA synthesis through its interaction with the DNA clamp PCNA (proliferating cell nuclear antigen) (18). Other histone chaperones including ASF1 and FACT (facilitates chromatin transcription) can also directly participate in S phase progression in all species from yeast to human (16,19). Both human ASF1 paralogs, ASF1a and ASF1b, isolated from nuclear extracts co-purify with the replicative helicase MCM2–7 and parental histones. This complex accumulates under replicative stress (14,20–21). ASF1a and ASF1b isolated from human cell extracts also associate with newly synthesized histones H3.1-H4, suggesting that this chaperone could participate in replication-coupled assembly pathways dedicated to both parental and new histones (16,21–24). Histone variant-specific chaperones like DAXX (dead domain-associated protein) and HJURP (Holliday Junction Recognition protein) are dedicated to the replication independent deposition of H3.3 and CENP-A, respectively (25,26), but both human ASF1 paralogs also interact with these variants and can participate in their deposition (27). Structural studies of ASF1, HJURP and DAXX provided insights into the way these histone chaperones recognize conserved features of histones and the molecular bases for specific recognition of histone variants (9–12,28–30).

The protein Mini Chromosome Maintenance 2 (MCM2) was proposed to play a central role in the processing of both parental and newly synthesized histones (14,20–21,31–32). MCM2 is positioned at the core of the replisome as a subunit of the replicative helicase MCM2–7 that unwinds DNA and separates the two strands of the double helix prior to the action of DNA polymerases. Together with the other subunits of the helicase (MCM3–7), MCM2 co-immunoprecipitates with histones H3-H4 from nuclear extracts in S phase in yeast and human cells (14,20,21,31–32). This complex accumulates during replicative stress and is enriched in parental histones in human cells (20). In human cytosolic cells extracts, MCM2 also co-immunoprecipitates with newly synthesized histones H3-H4 and with ASF1 (a and b) (14,20,21,32). Interestingly, this complex does not include the other replicative helicase subunits (MCM3–7), suggesting that independently from its role at the replication fork, MCM2 could also play a specific role as a histone chaperone. Former biochemical studies revealed that the MCM2 N-terminal tail directly binds histone H3 *in vitro* (33,34) and the binding properties were confirmed by mutagenesis in yeast (31). Consistent with a tight coupling between histone processing and DNA unwinding, deregulation of histone H3-H4 levels or MCM2 depletion lead to an accumulation of cells in S phase with slower DNA replication and impaired DNA unwinding (14,35). Interestingly, these defects are also observed after depletion of histone chaperones ASF1 and CAF-1 (14,35–36).

To gain further insights into the mechanisms involved in histone management during replication, we have solved the structure of the ternary complex between the histone-binding domain of MCM2 and the histones H3-H4. Free in solution, the MCM2 domain is mainly unfolded but, upon binding to histones, it wraps around the surface occupied by DNA in the nucleosome. The interaction with MCM2 induces a conformational change of helix αN of histone H3 and competes with the binding of H2B. We also found that MCM2 binds the tetrameric form of histones H3-H4 even at 0.5M NaCl. Upon addition of the conserved (1–156) N-terminal domain of the histone chaperone ASF1, the H3-H4 tetramer dissociates in favor of the formation of a stable quaternary complex comprising ASF1, H3, H4 and MCM2. Based on these results, we discuss possible models for new and parental H3-H4 histone management coupled to fork progression.

## MATERIALS AND METHODS

### Protein sample preparation

Recombinant *Drosophila melanogaster* histones H3-H4 (identical to human histones H3.2) were co-expressed with (His)_6_-dAsf1 from the pET28 plasmid (generous gift from R.N. Dutnall). The purification protocol was adapted from (37). *Escherichia coli* strain Lemo21 was grown over night in a ZY auto-inducible medium. Cells were collected by centrifugation, resuspended in lysis buffer (50 mM Tris-HCl pH8, 500 mM NaCl, 5% glycerol, 1% Triton X-100, 1 mM PMSF, 1 μM aprotinin, 0.25 mM DTT) and flash frozen in liquid nitrogen. After thawing, lysosyme was added at a final concentration of 1 mg/ml and cells were further lysed by sonication. Soluble (His)_6_-Asf1 was removed on a NiNTA column (Qiagen) equilibrated in the lysis buffer. The flowthrough was filtered with 0.22 μ filters to remove insoluble particles and loaded on a cation exchange Resource S column (GE Healthcare) equilibrated in 50 mM Tris-HCl pH8. Histones H3-H4 were eluted with a NaCl gradient in a buffer B containing 50 mM Tris-HCl pH8 and 2 M NaCl. After adjusting the salt concentration to 2 M NaCl, H3-H4 were then concentrated in a 3 kDa concentrator (Millipore). Histones were further purified by gel filtration with a superdex 200 column (GE Healthcare).

The three recombinant constructions of *Homo sapiens* MCM2 (MCM2 1–160, MCM2 63–153 and MCM2 69–138) were introduced in pETM30 plasmid fused with a (His)_6_-GST tag. *E. coli* strain BL21 (DE3) Gold was grown over night in standard LB medium. Cells were collected by centrifugation, resuspended in buffer A and flash frozen in liquid nitrogen. After thawing, lysosyme was added at a final concentration of 1 mg/ml and cells were further lysed by sonication. (His)_6_-tagged GST fusion proteins were immobilized on glutathione disulfide (GSH) agarose resin (Sigma) and eluted in a buffer containing 50 mM Tris-HCl pH8, 150 mM NaCl, 10 mM GSH. Fusion proteins were cleaved using a (His)_6_-tagged TEV protease (1% w/w). A HisTrap column (GE Healthcare) was used to trap the (His)_6_-tagged TEV and the (His)_6_-tagged GST. The flowthrough, containing MCM2, was finally concentrated in a concentrator (Millipore) with adapted cut-off in a buffer with 50 mM Tris-HCl pH8, 150 mM NaCl.

Recombinant *H. sapiens* ASF1a 1–156 was produced and purified using the same protocol as for MCM2 except that the HisTrap column was replaced by a nickel-nitrilotriacetic acid (NiNTA) column (Qiagen). The flowthrough was then loaded on an anion exchange column Resource Q (GE Healthcare) and ASF1 eluted using a gradient of an elution buffer with 50 mM Tris-HCl and 1 M NaCl. The elution buffer was replaced by 50 mM Tris-HCl pH8 storage buffer using an Amicon device (Millipore) and an YM10 regenerated cellulose membrane (Millipore).

### Nuclear magnetic resonance (NMR) experiments

For NMR experiments, production of ^15^N and ^13^C labeled MCM2 was obtained using the same protocol as described above except that the cellular culture was performed in a minimal medium supplemented with ^15^N ammonium chloride and ^13^C glucose (Eurisotop). NMR experiments were carried out on Bruker DRX-600 and DRX-700 spectrometers. For assignment experiments, purified ^15^N ^13^C MCM2 was concentrated to 90 μM and exchange in NMR buffer (50 mM Tris-HCl pH6, NaCl 1.5 M, 0.1 mM EDTA, 0.1 mM DSS, 0.1 mM NaN_3_, protease inhibitors (Roche), 10% D_2_O). Data for assignment of the backbone resonances were collected at 300°K using standard ^1^H-^15^N HSQC, ^15^N-edited NOESY-HSQC, TOCSY-HSQC, HNCA, HBHA(CO)NH, CBCA(CO)NH, HN(CA)CO, HNCO, HN(CO)CA, CBCANH and HN(CA)CO experiments. Proton chemical shifts (in ppm) were referenced relative to internal DSS and 15N and 13C references were set indirectly relative to DSS using frequency ratios (38). NMR data were processed using Topspin (Bruker) and analyzed using Sparky (T.D. Goddard and D.G. Kneller, UCSF). Values for random coiled chemical shifts used in the calculation of secondary Cα, Hα and CO shifts were taken from a study by (39–41). Interaction experiments were performed at 293°K adding a stock solution of 250 μM H3-H4 in a 10 mM pH7.5, 1.5 M NaCl to a 60 μM MCM2 sample 10 mM pH7.5, 1.5 M NaCl with a final MCM2:H3-H4 ratio of 1:3. 2D ^1^H-^15^N HSQC spectrum was recorded before and after histone addition.

### Crystallization and data collection

H3-H4-MCM2 (69–138) were mixed and the complex was purified by gel filtration (superdex 200, GE Healthcare) in a 50 mM Tris-HCl pH8, 1.5 M NaCl. Complex H3-H4-MCM2 (69–138) was concentrated to 2.5 mg/ml in a buffer 50 mM Tris-HCl pH8, 1.5 M NaCl. Crystals of the complex were grown by sitting drop vapor diffusion at 20°C against reservoir solution containing 0.1 M HEPES pH7, 21% PEG3000. Crystals were soaked in a cryo-protectant solution (0.1 M HEPES pH7, 21% PEG3000, 20% Glycerol) before being flash-frozen in liquid nitrogen.

Diffraction data were collected on the PROXIMA-1 beamline at the synchrotron SOLEIL (Saint Aubin, France) at a temperature of 100 K with high X-ray wavelength (1.6531 Å) to increase the sulfur anomalous signal. Diffraction images recorded with a PILATUS 6 M detector were processed using the XDS package (42). The best data set was obtained from a crystal of H3-H4-MCM2 (69–138) belonging to space group *R*32 which diffracted up to 2.9 Å resolution (Supplementary Table S2).

### Structure determination and refinement

The structure of H3-H4-MCM2 (69–138) complex was determined by molecular replacement using MOLREP (43) with the H3-H4 histones structure (chain A and B of PDB entry 1KX5) as model probe. Best solution contained one complex per asymmetric unit. Correctness of the solution was confirmed by the superposition of the full H3-H4 tetramer (chains A, B, E and F of PDB entry 1KX5) by applying the *R*32 2-fold crystallographic symmetry. Additional electron density could be modeled and attributed to the MCM2 peptide from amino acids G69 to D121. Sulfur atom positions were confirmed by analyzing the anomalous difference Fourier maps calculated with the AnoDe program (44). This allowed to confirm the position of the two methionine in the MCM2 69–121 traced sequence (Supplementary Figure S5).

Model building was performed with Coot (45) and structure refinement was achieved with BUSTER version 2.9 (Cambridge, United Kingdom). Final refinement statistics are presented in Supplementary Table S2. Structure representations presented in all the figures were drawn with PYMOL software (Schrödinger).

### Analytical gel filtration experiments

H3-H4, MCM2 (69–138) and hASF1a were analyzed both individually and in combination. These proteins were run alone at 20 μM or protein complexes were pre-formed with the molar ratios (1:1:1:1) and incubated the time indicated on the figure on ice before loading the sample on the column. For each run, 100 μl of sample was injected on a Superdex 200 Increase 10/300 GL column (GE) at a flow rate of 0.5 ml/min. The column was equilibrated with the buffer (Tris 50 mM pH 8, NaCl 0.5M) before loading and the proteins were eluted with the same buffer. Fractions were collected for each run and analyzed by sodium dodecyl sulphate-polyacrylamide gel electrophoresis (SDS-PAGE) (18% acrylamide gel, samples stained with Coomassie Blue). Quantification of the histone fraction bound to MCM2 only (peak 1) or associated with both ASF1 and MCM2 (peak 2) was obtained by integrating the peak area at each time point and dividing by the molar extinction coefficient of each complex considering histone dimers in both cases. Quantitative results are reported as percentages (Figure 3C).

**Figure 1.**
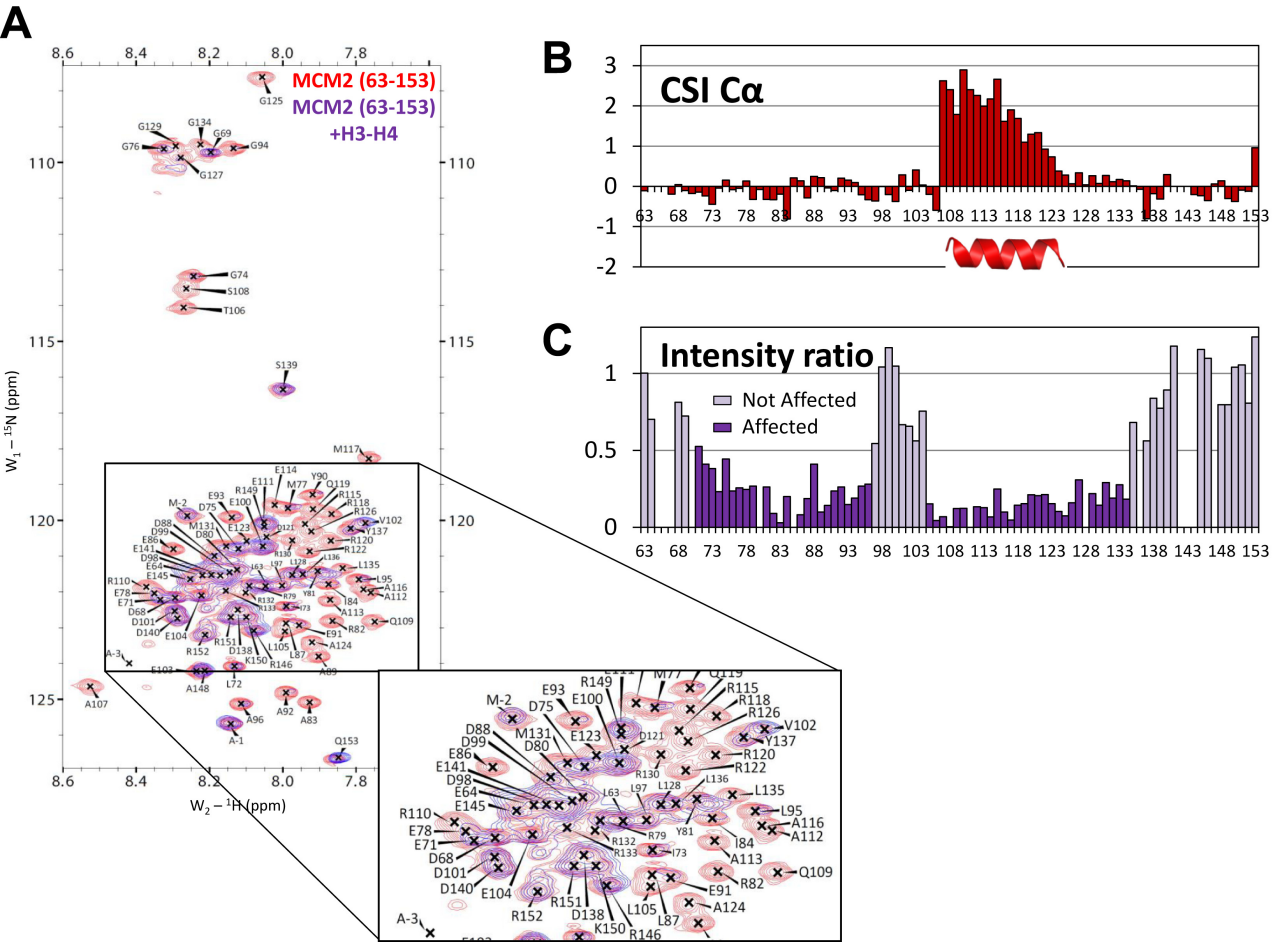
The histone H3-H4 interacting domain of MCM2 (A) Overlay of the ^1^H-^15^N HSQC spectra of MCM2 (63–153) free (red) and in excess of full-length histones H3**–**H4 (purple) (the spectra were recorded at 20°C, in a Tris 10 mM buffer pH 8, 1.5 M NaCl). Signals disappearing in the bound form report for residues of MCM2 involved in the interaction with histones. (B) Histogram of the ^13^Cα chemical Shift Index (CSI) plotted with Wishart's reference sets for the unfolded state (see Methods for details) along the sequence of free MCM2 (62–153) highlights the propensity of the residues 107–121 to adopt a helical conformation (positive CSI > 1.0). (C) Intensity ratios of the ^1^H-^15^N HSQC signals between bound and free MCM2 (63–153) indicate that residues 72–134 are involved in the interaction with histones H3-H4 except for the 97–104 segment.

**Figure 2.**
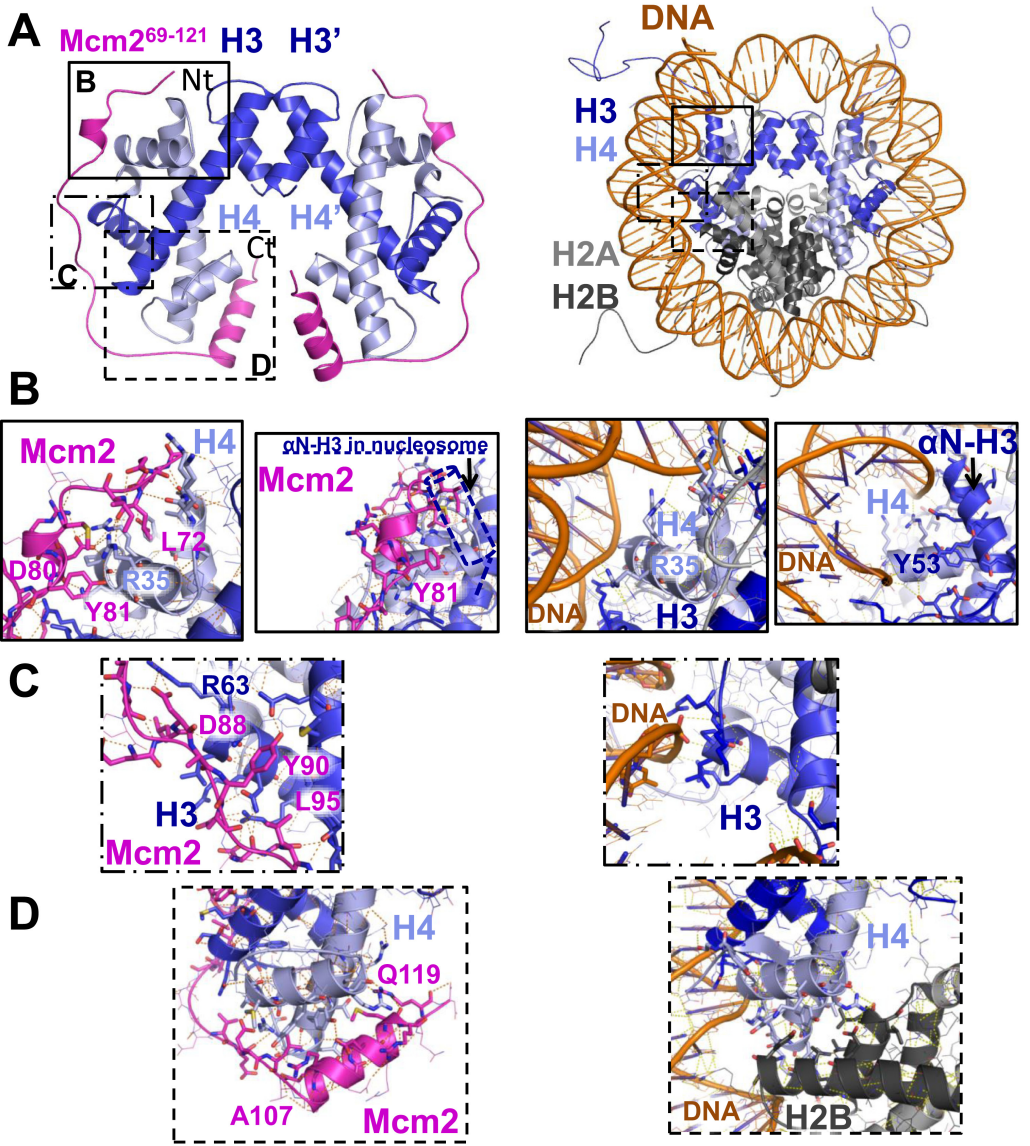
Structure of the ternary complex containing two MCM2 histone-binding domains (69–121) bound to the histone H3-H4 tetramer. (A) Ribbon representation of the MCM2^69^–^121^−H3^23^–^100^−H4^57^–^134^ complex compared to the structure of the nucleosome (PDB code 1KX5 (1)) on the left and right panels, respectively. MCM2, H3 and H4 are in magenta, dark blue and light blue, respectively. H2A, H2B and DNA (right panel) are in light gray, dark gray and brown, respectively. H3′ and H4′ labels indicate the location of the second histones heterodimer. Three boxes in solid, dot-dashed and dashed lines (shown as close-up views in panels B–D) specify the zones anchoring MCM2 to the histones (left panel) and corresponding regions are boxed in right panel. In the close-up views, residues side-chain of MCM2 taking part to the interaction with H3−H4 heterodimer are shown as sticks while other side-chains are shown as lines. (B) Interactions made by MCM2 residues Leu72, Asp80 and Tyr81 with histone H4 helices. A rotation of the view shows that MCM2 helix (77–81) is incompatible with the binding of the αN-helix of histone H3 as in the nucleosome (right). No density for the αN residues of histone H3 could be observed in the structure of the MCM2−H3−H4 complex. (C) Close-up view of interactions made by MCM2 residues Asp88, Tyr90 and Leu95 with histone H3 helices. (D) The last helix (107–119) in the MCM2 histone-binding domain interacts with histone H4 (left panel) in the same region as the one involved in the binding of histone H2B in the entire nucleosome structure (right panel).

**Figure 3.**
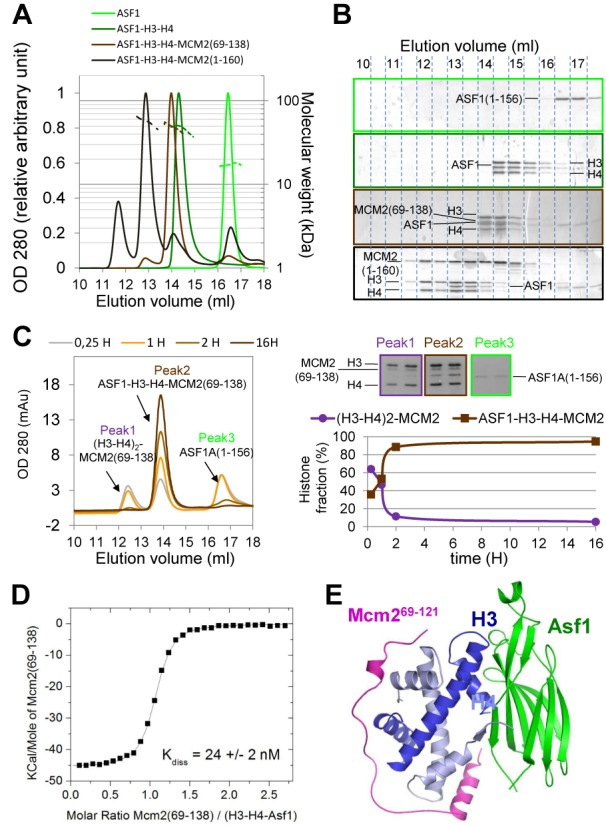
Biophysical characterization and modeling of the quaternary complex formed by MCM2, ASF1 and the histone H3−H4 heterodimer. (A) Sec-MALS analysis at 20°C, in a Tris 50 mM buffer pH 8, 0.5 M NaCl of human ASF1a (1–156) (in green), ASF1a (1–156)-H3-H4 in dark green, ASF1a (1–156)-H3-H4-MCM2 (69–138) in light brown and ASF1a (1–156)-H3-H4-MCM2 (1–160) in dark brown. Relative optical density at 280 nm was plotted in arbitrary units in continuous lines as a function of the elution volume. The calculated molecular mass is reported as dashed line in the corresponding color with the secondary scale on the right. (B) SDS-PAGE analysis of Sec-MALS fractions for the different samples collected in A with a color code as in A. (C) Time course analysis of quaternary complex formation by gel filtration. Equimolar samples of the proteins ASF1, H3-H4 and MCM2 (69–138) were mixed at time zero and injected in the column at the indicated time. Composition of the three peaks is analyzed by SDS-PAGE on the right panel together with quantification of the histone fraction bond to MCM2 only or to ASF1and MCM2 over time. (D) Dissociation constant (*Kdiss*) of MCM2 (69–138) in complex with the preformed H3−H4−ASF1a (1–156) ternary complex as determined by ITC in 0.5 M NaCl, pH 8, at 20°C. (E) Model of the structural arrangement of the MCM2-H3-H4-ASF1 quaternary complex obtained by superimposing the structure of H3−H4 dimer in the H3−H4−ASF1a (1–156) ternary complex (PDB code 2IO5 (12)) with its structure in the MCM2 (69–121)-H3-H4 complex. ASF1 and MCM2 can accommodate around the histone H3−H4 heterodimer without any clash.

### Size-exclusion chromatography with multi-angle laser light scattering (Sec-MALS)

For molar mass determination, purified proteins were analyzed using an high pressure liquid chromatography (HPLC)-MALS system. Note that 200 μg of protein solution were injected on Superdex 200 10/300 gl Increase (GE Healthcare) equilibrated in 50 mM Tris pH 8.0 supplemented by 0.5 or 1.5 M NaCl at a flow rate of 0.5 ml/min. Separation and ultraviolet detection were performed using Shimadzu HPLC system, light scattering was monitored using mini DAWN TREOS system (Wyatt Technology) and concentration was measured by Optilab T-rEX differential refractometer (Wyatt Technology). Molar masses of proteins were calculated using ASTRA 6.1 software (Wyatt Technology) and *dn*/*dc* values were determined following the equation (46):

\begin{equation*} \left( {\frac{{dn}}{{dc}}} \right)_p = \left( {\frac{{dn}}{{dc}}} \right)_{p,{\rm water}} - \bar \upsilon _p (n - n_{{\rm water}} ) \end{equation*}

where water refers to an aqueous solution of protein of 0.183 ml/g and $\bar \upsilon _p$ is the partial-specific volume of proteins determined by using SEDNTERP (T. Laue, Royal Society of Chemistry, Cambridge).

### Analytical ultracentrifugation (AUC)

All sedimentation velocity and sedimentation equilibrium experiments were performed on an analytical ultracentrifuge XLA (Beckman Coulter, Palo Alto, USA) with an An-60 Ti rotor at 20°C. Sedimentation velocity experiments were done in two-channel 12 mm path-length aluminum centerpieces. Note that 360 μl of H3-H4, MCM2 and H3-H4-MCM2 samples in a buffer containing 50 mM Tris, 1.5 M NaCl, pH 8.0, were centrifuged at 50 000 revolutions per minute (rpm), absorbance at 280 nm was from 0.5 to 1.2. Sedimentation profiles were collected every 5 min. Sedimentation velocity boundaries were analyzed using SEDFIT software (47).

Sedimentation equilibrium experiments were performed by loading a sample of protein mixture (110 μl) in two-channel 12-mm path-length Epon charcoal-filled centerpiece. Three different concentrations of H3-H4-MCM2 were loaded and centrifuged at successive speeds of 10 000, 13 000 and 17 300 rpm until sedimentation equilibrium was achieved. Sedimentation equilibrium data were analyzed using SEDPHAT (47).

The buffer density and viscosity at 20°C were determined by SEDNTERP software (T. Laue, Royal Society of Chemistry, Cambridge), partial specific volumes for H3-H4 and MCM2 were calculated based on the amino acid composition using this program.

### Isothermal titration calorimetry

All IsoThermal Calorimetry (ITC) experiments were performed in a VP-ITC titration calorimeter (MicroCal, Northampton, MA, USA). Before loading, the protein solutions were extensively degassed. The protein complex solution, H3-H4 or H3-H4-ASF1, was placed in the calorimeter cell and the titrant, MCM2 solution, was loaded into the rotating injector syringe at ∼10 × the protein complex concentration. For the ITC experiment MCM2 with H3-H4 complex, both were dialyzed overnight at 4°C in the same buffer (Tris 50 mM pH8, NaCl 1.5 M). For the ITC experiment involving the complex H3-H4-ASF1, the ternary complex was purified in the appropriate buffer (Tris 50 mM pH8, NaCl 0.5 M) after a size exclusion chromatography step. MCM2 concentration was then adjusted and the protein was dialyzed against the same buffer. After equilibrating the cell at 20°C, the rotating syringe (307 rpm) injected 6 μl aliquots of MCM2 solution into the protein complex solution at intervals of 280 s until saturation was observed. Raw ITC data were analyzed with the Origin 7.0 software (Origin­Lab). A one set of sites model was fitted to the sigmoidal curves obtained and the following parameters were determined: stoichiometry (*N*), association constant (*KA*) (*KD* was deduced as *KD* = 1/KA) and the change in enthalpy (Δ*H*). The values of the standard molar Gibbs energy change (Δ*G*) and standard molar entropic contribution (*T*Δ*S*) were then calculated using the relationships Δ*G* = - *RT*ln(*KA*) = *T*Δ*S* - Δ*H*, where *R* is the gas constant (8.314472 J.K^-1^mol^-1^) and *T* is the temperature in kelvin (293.15 K). All ITC experiments were performed in duplicate.

## RESULTS

### The histone H3-H4 interacting domain of MCM2

Previous studies showed that the N-terminal region of MCM2 is implicated in histone binding (31,33–34). This region does not belong to the AAA ATPase domain present in all MCM2–7 subunits but corresponds to a specific N-terminal extension present in all MCM2 proteins from *P. falciparum* to humans (Supplementary Figure S1). We purified a uniformly ^15^N labeled sample of human MCM2 (1–160) as a recombinant protein in *E. coli* and analyzed its structure by standard heteronuclear NMR techniques. Spectral dispersion of NH resonances demonstrates that this fragment is mainly disordered in solution (Supplementary Figure S2 in black). Addition of unlabeled full-length histones H3-H4 led to the vanishing of a subset of HSQC resonances (Supplementary Figure S2A in blue) confirming the capacity of this region to interact with histones H3-H4 *in vitro*. A subset of resonances remained unchanged after histone addition showing that only a subfragment of MCM2 (1–160) is implicated in the interaction. Based on the sequence conservation profile of MCM2 (1–160), a shorter MCM2 (63–153) fragment was designed (Supplementary Figure S1). All resonances of the short MCM2 fragment (63–153) superimpose well with those of MCM2 (1–160) in the HSQC spectrum (Supplementary Figure S2B in red and black, respectively). In the presence of histones H3-H4, exactly the same set of resonances is affected between MCM2 (1–160) and MCM2 (63–153), showing that the histone binding region can be restricted to the conserved domain. Resonance assignments of MCM2 (63–153) were achieved using a uniformly ^15^N-^13^C sample by standard heteronuclear techniques (Figure 1A). Chemical shift indexes of alpha carbons (Figure 1B) and all other backbone nuclei (Supplementary Figure S3) reveal the presence of a well-defined residual helix spanning residues 107–122. Upon addition of full-length recombinant H3-H4 histones, two subsets of resonances from residues 71–96 and 104–133 vanish and delineate two segments of MCM2 in contact with histones (Figure 1C), one of them including the residual helix (107–122) identified above. In contrast, residues 63–69, 97–104 and 135–153 remain unchanged upon addition of histones (Figure 1A and C). The histone binding region could thus be further restricted to the (69–138) segment. Affinities of MCM2 (1–160) and MCM2 (69–138) for histones were measured using ITC with dissociation constants of 0.19 ± 0.02 μM (Supplementary Figure S4A, Supplementary Table S1) and 0.24 ± 0.1 μM (Supplementary Figure S4B, Supplementary Table S1), respectively, confirming that the shorter region recapitulates all interactions made by the MCM2 N-terminal region with histones. A significant enthalpy change Δ*H* of −34.8 ± 0.5 kcal mol^−1^ was measured for MCM2 (1–160) (−35.1 ± 1.2 kcal mol^−1^ for MCM2 (69–138)), likely arising from electrostatic interactions between the acidic character of MCM2 N-terminus and the basic histones. However, the effect of salt could not be further quantified due to limited solubility of histones at low salt concentrations (Supplementary Figure S11). Balancing the large enthalpic contribution, a highly unfavorable entropy Δ*S* of −26.3 ± 0.5 kcal mol^−1^ was estimated for MCM2 (1–160) (−26.7 ± 0.2 kcal mol^−1^ for MCM2 (69–138)), reflecting a significant loss of conformational entropy upon histone binding together with a low contribution of the hydrophobic effect (48). In conclusion, we defined a histone binding region in the N-terminal segment of MCM2, MCM2 (69–138). This segment is largely unfolded in solution, with a residual (107–122) helix. It forms a stable complex with histones H3-H4 at 1.5 M NaCl.

### Structure of H3-H4-MCM2

The complex reconstituted by mixing MCM2 (69–138) with full-length histones H3-H4 was crystallized and diffraction data were recorded at 2.9 Å resolution (Supplementary Table S2). The structure could be solved by molecular replacement using the structure of one histone H3-H4 dimer as outlined in Materials and Methods (Supplementary Figure S5). One molecule of each protein H3, H4 and MCM2 is present in the asymmetric unit. After refinement, electron density is observed for residues S57 to R134 of histone H3, R23 to F100 of histone H4 and G69 to D121 of MCM2 (Figure 2A). The crystallographic symmetry reconstitutes a tetramer of histones H3-H4 which closely resembles that of the nucleosome structure (Cα RMSD of 1.12 Å) (Figure 2A and B) showing that the binding of MCM2 is compatible with the formation of the H3-H4 tetramer. The MCM2 fragment (69–121) wraps around the H3-H4 dimer complex and follows the position of DNA in the nucleosome so that it clearly competes with the binding of DNA. MCM2 residues G69-L97 and V102-R120 have extensive contacts with both histones H3 and H4 and adopt mainly an extended conformation interrupted by two helical segments: one helical turn involving residues M79-Y81 and a well-defined helix from residues A107 to R118. The latter matches the residual helix previously observed in solution with a C-terminal fraying end (Figure 1B). MCM2 residues D98-D101 are not directly in contact with histone residues in the asymmetric unit but establish contacts with symmetrical histone molecules. Since these residues were not found in interaction with histones in the NMR experiments (Figure 1C), these contacts with symmetrical histones most probably stabilize the crystal packing but do not correspond to specific interacting regions. Residues 122–138 are not observed in the electron density suggesting that they remain mobile although residues 122–135 HSQC resonances were perturbed upon addition of histones. They probably establish less specific contacts than the MCM2 (69–121) region. Consistent with the amino acid composition of both H3-H4 and MCM2 (69–121) moieties, the highly polarized electrostatic potential confirms that the complex is stabilized by charged attractive interactions (Supplementary Figure S6) fully compatible with the large change in binding enthalpy as measured by ITC. The most intense electrostatic complementarity follows the trace of DNA binding while the C-terminal helix of MCM2 (69–121) interacts with a more apolar groove at the surface of histone H4.

As shown from the multiple sequence alignment between remotely related species (Supplementary Figure S7), the most conserved positions correspond to residues establishing important interactions at the interface between MCM2 and histones H3-H4. From the N- to C-terminus of MCM2, three major anchoring sites binding to histones can be discriminated. The first region corresponds to MCM2 residues G69-Y81. A network of polar and charged contacts are formed with the side-chains of three arginine residues of histone H4 (R35, R36 and R39) that establish close contacts with the DNA in the nucleosome (Figure 2B). This segment of MCM2 binds the α3 helix of histone H4 so that it competes with the docking of αN helix of histone H3 (Figure 2B). In particular, the Y81 side-chain docks exactly into the same position as Y53 in the αN helix of histone H3. The αN helix was previously proposed to mediate the interaction with MCM2 (34), but it is not observed in our crystal structure (and we could not detect any interaction with a histone H3 (27–63) peptide corresponding to the isolated αN helix, unpublished data). This helix is most probably displaced upon MCM2 binding. The conserved position corresponding to the anchoring residue Y81, was mutated in yeast and showed reduced binding to histones (31), confirming its key contribution to the interaction.

The second MCM2 region establishing tight contacts with histones is centered on Y90 and spans residues E86-A96 (Figure 2C). Y90 docks into an apolar cavity at the interface between α1 and α2 helix of histone H3 while MCM2 chain wraps around the DNA binding region including histone H3 R63. The highly conserved position corresponding to residue Y90 was also mutated in yeast and was shown to induce a severe decrease in binding to histones (31). A striking similarity can be noted in the way that other histone H3-H4 chaperones, DAXX and HJURP, bind the apolar cavity recognized by Y90 and L95 residues, although they share no homology with MCM2 (Supplementary Figure S8A–C, left and middle panels). Both histone partners combine aromatic and aliphatic side-chains to ensure tight binding to histones (DAXX Y222 with L210 and HJURP W66 with V50) (28,29).

The third MCM2 region in close contact with histones H3-H4 encompasses residues V102-R121 (Figure 2D, left panel). The first part of this segment adopts an extended conformation and competes with DNA binding and the second part corresponds to a helix that docks on histone H4 in a more apolar groove. This region competes with histone H2B binding (Figure 2D, right panel) and is also targeted by the histone chaperone DAXX in a similar manner (Supplementary Figure S8A and B, right panels). MCM2 (69–121) does not contact the residues which differ between the three histone variants H3.1, H3.2 and H3.3 except V89 (I89 in H3.3). This mutation is conservative and is predicted not to modify MCM2 histone binding. This result is consistent with the fact that MCM2 helicase does not discriminate between these three canonical histone H3 variants (32). Altogether, the binding mode of MCM2 and histones H3-H4 is reminiscent of the way histone chaperones bind their target, shielding surfaces otherwise buried by other histones or DNA in the nucleosome. By preventing these surfaces from spurious interactions, MCM2 may be considered as a genuine histone assembly chaperone.

### MCM2 binds the H3-H4 tetramer at 1.5 M and 0.5 M NaCl and prevents histone aggregation at 0.5 M NaCl

In order to confirm the stoichiometry of the complex in solution, we used gel filtration experiments coupled to multiple angle light scattering (Sec-MALS) and AUC. As observed by NMR, we confirmed that the MCM2 (1–160) fragment forms a stable complex with recombinant full-length H3-H4 histones at high salt concentration (1.5 M NaCl) (Supplementary Figure S9A and B). The molecular mass of the complex as measured by Sec-MALS is 69.0 ± 0.3 kDa (Supplementary Figure S9A), and by AUC 77.67 ± 2.1 kDa for the hydrated complex and 71.5 kDa for the anhydrous complex (Supplementary Figure S10A–C) shows that, in these conditions, histones H3-H4 form a tetramer in interaction with only one MCM2 (1–160) molecule (Table 1). The same stoichiometry, one histone tetramer binding to one MCM2 (69–138) molecule, was measured for the complex with the shorter MCM2 fragment used for crystallization (Supplementary Figure S11A and B, Table 1). This complex differs from that observed in the crystal structure (two MCM2 molecules per histone H3-H4 tetramers).

**Table 1.** Mass measurements by Sec-MALS

Complex^a^	Molecular ratio 1^b^	Expected mass 1^b^	Molecular ratio 2^b^	Expected mass 2^b^	Molecular ratio 3^b^	Expected mass 3^b^	Measured mass at 0.5M NaCl^c^	Measured mass at 1.5M NaCl^d^
H3-H4	1:1	26.7	**2:2**	**53.4**	-	-	**ND**	**54.6** ± **0.4**
MCM2_L_	**1**	**16.7**	2	33.4	-	-	**16.7** ± **0.3**	**17.1** ± **0.4**
H3-H4-MCM2 _L_	1:1:1	43.4	**2:2:1**	**70.1**	**2:2:2**	**86.8**	**86.8** ± **2.3**	**69.0** ± **0.3**
ASF1	**1**	**17.9**	2	35.8	-	-	**17.1** ± **0.3**	**18.7** ± **0.7**
H3-H4-ASF1	**1:1:1**	**44.7**	2:2:1	71.5	2:2:2	89.4	**50.3** ± **0.3**	**43.9** ± **0.3**
H3-H4-MCM2 _L_-ASF1	**1:1:1:1**	**61.3**	2:2:1:1	88.0	2:2:2:2	122.6	**59.5** ± **0.2**	**ND**
MCM2_S_	**1**	**6.7**	2	13.4	-	-	**7.3** ± **0.1**	**7.9** ± **0.3**
H3-H4-MCM2_S_	1:1:1	33.5	**2:2:1**	**60.2**	**2:2:2**	**66.9**	**73.2** ± **3.8**	**61.1** ± **0.7**
H3-H4-MCM2_S_-ASF1	**1:1:1:1**	**51.3**	2:2:1:1	78.1	2:2:2:1	102.7	**49.9** ± **0.2**	**ND**

^a^MCM2_L_ indicates MCM2 (1–160), MCM2_S_ indicates MCM2 (69–153), ASF1 indicates ASF1a (1–156).

^b^Calculated ratio and molecular masses that correspond to the measured mass are indicated in bold.

^c^Buffer conditions: Tris 50 mM pH8, NaCl 0.5 M, ND : not determined.

^d^Buffer conditions: Tris 50 mM pH8, NaCl 1.5 M, ND : not determined.

High salt concentration has previously been shown to stabilize the histone H3-H4 tetramer, while lowering the salt concentration destabilizes the tetramer in favor of the dimer. This equilibrium is concentration and pH dependent (49,50). In the conditions we used for our biophysical analysis (Tris 50 mM, pH 8 and various NaCl concentrations), we observed a low solubility of free H3-H4 histones (even for histone concentrations as low as 20 μM) with an irreversible aggregation below 0.7 M NaCl. Upon addition of MCM2 (1–160) or MCM2 (69–138), the solubility of histones was restored at 0.5 M NaCl (Supplementary Figure S11C and D). In this condition, we measured a mass of 86.8 ± 2.3 kDa for the H3-H4-MCM2 (1–160) complex and 73.2 ± 3.8 for the H3-H4-MCM2 (69–138) complex, which is compatible with a histone H3-H4 tetramer interacting with two molecules of MCM2 (Table 1) as observed in the crystal structure.

Altogether, our results show that, in solution, histones H3 and H4 bind MCM2 as a tetramer as observed in the crystal structure, even at 0.5 M NaCl concentration where histones alone were mainly insoluble. In the later condition, MCM2 prevents histones from aggregation. The number of bound MCM2 molecules depends on salt concentration. At 0.5 M NaCl two MCM2 molecules per tetramer are observed in solution and in the crystal structure. At high salt (1.5 M NaCl), the complex is partially destabilized as expected for a complex largely stabilized by electrostatic interactions, but the binding affinity of 0.2 μM remains significant. Interestingly, only one molecule of MCM2 dissociates from the histone tetramer and the complex is asymmetric with one MCM2 molecule per histone tetramer. This structure might be related to that formed at the replication fork where only one MCM2 molecule is present.

### Binding of the H3-H4-MCM2 complex to the histone chaperone ASF1 dissociates the tetramer

ASF1 is a histone chaperone found to escort and stabilize histones H3-H4 *in vitro* and *in vivo* in all studied eukaryotes (22,24,51) with some variations between species: ASF1 deletion is lethal in *S. pombe* and chicken DT40 cells but not in *S. cerevisiae*. In vertebrates, two paralogous genes exist, ASF1a and ASF1b, that coevolved under subfunctionalization scenarios (52). In human cells, both proteins ASF1a and ASF1b, co-immunoprecipitate with MCM2. This complex was proposed to be mediated by histones H3-H4 (14,20), but so far this complex has not been reconstituted *in vitro*. As MCM2 stabilizes the tetramer and ASF1 dissociates the tetramer and binds the H3-H4 dimer, we wondered which state of histones, tetrameric or dimeric, could be observed in the reconstituted quaternary complex. The conserved domain of human ASF1a (1–156) was shown to be sufficient for histone binding (9) and was thus used in the reconstitution experiment. In a preliminary experiment, we verified that ASF1a (1–156) dissociates the H3-H4 tetramer and binds the H3-H4 dimer with the ASF1:H3:H4 1:1:1 stoichiometry by Sec-MALS (Figure 3A and B, Table 1). The four proteins H3-H4, ASF1a (1–156) and MCM2 (69–138) were then mixed and the formation of complexes was monitored by gel filtration (Figure 3C). Just after mixing the proteins, three species corresponding to different complexes were separated by the column: the (H3-H4)_2_-MCM2 (69–138) (Peak 1), free ASF1a (1–156) (Peak 3) and a new complex comprising the four proteins, ASF1a (1–156), H3-H4 and MCM2 (69–138) (Peak 2) (Figure 3C). Over time, the intensity of the peak corresponding to the quaternary complex (Peak 2) increased while the two other peaks (1 and 3) decreased. Thus, upon mixing, tetrameric histones first bind MCM2 and dissociate in a second step to form the quaternary complex. Quantification of the histone fraction bound to MCM2 only (Peak 1) versus the histone fraction associated to both proteins ASF1 and MCM2 (Peak 2) reveals that 60% of histones is first trapped in a complex with MCM2 before associating with ASF1 in our first experimental point (around 15 min). This fraction drops down to about 10% after 2 h and 6% after overnight incubation (Figure 3C). In contrast, when the histones were previously incubated with ASF1, the complex with MCM2 formed immediately without any (H3-H4)_2_-MCM2 intermediate complex. A binding affinity of 24 ± 2 and 280 ± 30 nM between MCM2 (69–138) and ASF1-H3-H4 could be measured by ITC at 0.5 and 1.5 M NaCl, respectively (Figure 3D and Supplementary Figure S12). Interestingly, we observe that the affinity of MCM2 for histones is not modified by ASF1 binding (compare the affinity of 280 ± 30 nM measured with ASF1with that of 240 ± 100 nM measured in its absence, Table 1), suggesting that both MCM2 and ASF1 bind histones independently. The molecular masses of the quaternary complexes were measured by Sec-MALS with values of 59.5 ± 0.2 kDa for the complex with MCM2 (1–160) and 49.9 ± 0.2 kDa with MCM2 (69–138) (Figure 3A and B and Table 1). These values are compatible with a stoichiometry of 1:1:1:1 for both ASF1:H3:H4:MCM2 complexes showing that the oligomeric state of histones is dimeric in the quaternary complex as in the ASF1-H3-H4 complex. Based on these data, a model for the structure of the ASF1-H3-H4-MCM2 quaternary complex can be inferred from the superimposition of histones dimers as organized in both structures of H3-H4-MCM2 (this study) and H3-H4-ASF1 complexes (12) without generating any steric hindrance (Figure 3E). This model highlights that MCM2 and ASF1 protect a large fraction of the surface accessible in the isolated dimer of histones H3-H4. Strikingly, almost all these regions match the sites of H3-H4 involved in the interaction with other constituents of the nucleosome (Supplementary Figure S13) except a single surface patch corresponding to the contact region with DNA in the Dyad region (H3 115–121). Upon MCM2 and ASF1 binding, these regions are protected against unwanted interactions, suggesting that both proteins could act as chaperones of H3-H4 at the replication fork.

## DISCUSSION

In this study, the structure of the conserved N-terminal tail of the helicase MCM2 in complex with histones H3-H4 revealed how a helicase can interfere with the nucleosome structure by competing with both the binding of DNA and histone H2B (Figure 2A and Supplementary Figure S13). We found that the MCM2 N-terminal tail is intrinsically disordered and folds upon histone binding. The binding interface involves mainly electrostatic interactions between acidic residues and histone basic residues involved in DNA binding. In addition, conserved hydrophobic and aromatic residues in MCM2 dock into apolar cavities. These cavities are also recognized by other histone chaperones or other histone regions interacting within nucleosomes (Supplementary Figure S8).

In the absence of ASF1, MCM2 interacts with the tetrameric state of histones H3-H4 in all studied conditions. At 0.5 M salt concentration, a condition that was previously shown to favor tetramer dissociation (50) and for which we observed a low solubility of histones H3-H4, MCM2 prevents histone aggregation and associates with the tetramer in a symmetrical complex (H3-H4-MCM2 2:2:2). Nevertheless, the crystal structure revealed that MCM2 does not bridge the two interacting histone dimers. Rather, each MCM2 molecule interacts with one H3-H4 dimer, and the tetramer is obtained by crystallographic symmetry operations. Consistent with a MCM2 binding site restricted to one histone dimer, the affinity of MCM2 for tetrameric histones (H3-H4)_2_ at 1.5 M NaCl (0.24 ± 0.1 μM) is comparable to that of MCM2 for dimeric histones in ASF1-H3-H4 complexes in the same buffer conditions (0.28 ± 0.03 μM) suggesting that MCM2 does not preferentially recognize the tetrameric form at the expense of the dimer stabilized by ASF1. The protection against aggregation observed at 0.5M NaCl concentration might results from an indirect electrostatic compensation of repulsion forces that could drive tetramer dissociation at this salt concentration. In agreement with an important contribution of electrostatics in stabilizing the complex, its binding affinity is sensitive to salt concentration (∼200 nM at 1.5 M NaCl and ∼20 nM at 0.5 M NaCl). Interestingly, this binding affinity remains modest considering the large surface buried by the interaction of MCM2 with histones H3-H4 (3678 Å^2^) and is weaker than the affinity of ASF1 for H3-H4 as measured in similar conditions (∼0.2 nM, ∼1360 Å^2^ buried surface (12,53)) or than that of the histone chaperone CAF-1 for histones H3-H4 (∼5 nM, unknown surface) (50,53–54). The modest affinity of MCM2 compared to its binding surface can be explained by its unfolded nature implying large conformational entropy cost upon binding. Such thermodynamic behavior was also described for the unfolded histone chaperone Chz1. This H2AZ-H2B chaperone was shown to fold upon histone binding with mainly electrostatic attractive interactions. The association rate constant of the complex was high (100 times faster than diffusion) and consequently, the Chz1-histones complex life time was shown to be remarkably short (∼0.1 s) (55,56). MCM2 could similarly attract histones through long distance attractive electrostatic interactions that have a short life time, allowing other partner to capture histones. This property could explain why, upon mixing H3-H4, MCM2 and ASF1, the MCM2-(H3-H4)_2_ complex is kinetically favored before the formation of the more stable quaternary complex ASF1-(H3-H4)-MCM2 (Figure 3C). Remarkably, the large majority of the H3-H4 histone surface interacting with nucleosome components is buried by ASF1 and MCM2 suggesting that both proteins could prevent histones from other unwanted interactions. In this respect, MCM2 could be considered, like ASF1, as a chaperone preventing aggregation or unwanted toxic non-specific interactions *in vivo*.

In human cells, it has been proposed that, in combination with other histone chaperones and remodelers, MCM2 and ASF1 play a central role in histone assembly coupled to DNA replication in pathways dedicated to both newly synthesized and parental histones (14,20–21,31–32). Our results provide further insights into the nature of interactions that could take place between these actors.

**Figure 4.**
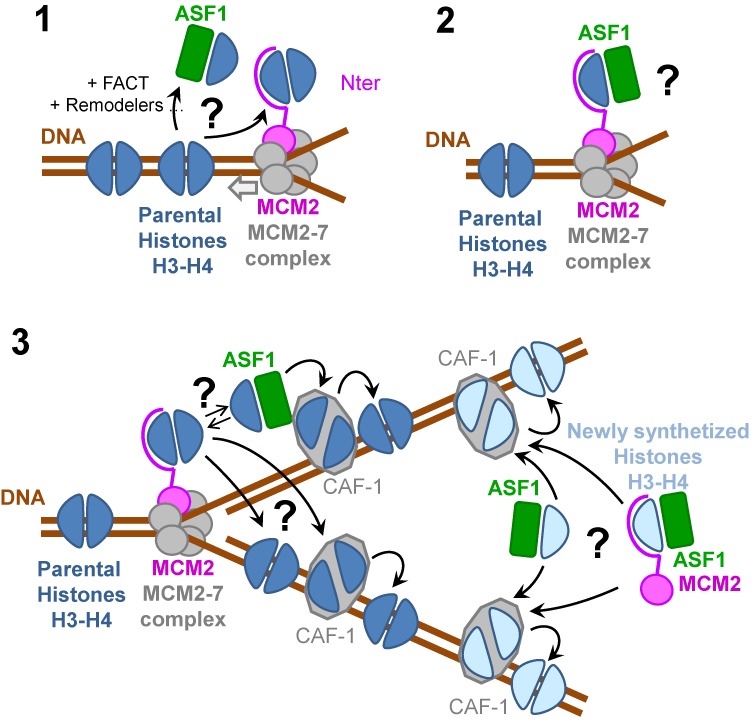
Models for the role of the ternary (H3-H4)_2_-MCM2 complex and the quaternary ASF1-H3-H4-MCM2 complex in handling histones during replication. (1) Several pathways can be envisaged for parental histone dissociation upstream the replication fork. After removal of H2A-H2B by dedicated chaperones (not represented on the figure), histone chaperones dedicated to H3-H4 could dissociate histones from DNA. Alternatively, the replicative MCM2–7 helicase could also destabilize interactions of parental histones H3-H4 with DNA via its mechanical force. The (H3-H4)_2_-MCM2 ternary complex could then be transiently formed and protecting from re-association with DNA upstream the replisome machinery. (2) The quaternary Asf1–(H3-H4)–MCM2 complex observed in our study could constitute the next intermediate step further protecting and destabilizing the H3-H4 complex. (3) Parental histone reassembly could proceed by different mechanisms; the tetramer captured by MCM2 the N-terminus of MCM2 could be directly deposited on DNA without splitting or this histone tetramer it could be directly transferred to the assembly chaperone CAF-1. Alternatively, histone tetramers split by ASF1 could be reassembled by CAF-1 upon deposition on DNA. Besides, the human pathway dedicated to newly synthesized histones was also shown to involve ASF1 and CAF-1. The major histone fraction associated with MCM2 carries the specific modifications of newly synthesized histones suggesting that the quaternary complex could also transfer histones to CAF-1 for further assembly. All presented pathways remain hypothetical, may not be conserved in all species and may be restricted to some regions of the chromatin.

The pathway dedicated to parental histones implies nucleosome disassembly upstream of the replication fork and nucleosome reassembly downstream of the fork after DNA duplication. It is generally assumed that H2A-H2B dimers are bound by dedicated chaperones, dissociated before histones H3-H4 and reassembled after histones H3-H4 (16). Many uncertainties remain concerning the histone H3-H4 disassembly process. This dissociation has been associated with the action of several histone chaperones including FACT, ASF1 and/or histone remodelers (15,16) (Figure 4, panel 1). Histones H3-H4 could also be processed by an additional pathway that would not imply any external chaperone. The mechanical force of the MCM2–7 helicase could be sufficient to destabilize the DNA-(H3-H4)_2_ complex (tetrasome), the N-terminal region of MCM2 would then be in close proximity to capture the histones H3-H4 tetramer and would compete with DNA reassembly (Figure 4, panel 1). In this model, the complex formed should correspond to the asymmetrical complex (H3-H4-MCM2 2:2:1) because only one copy of the helicase is present at the fork (57). Our data are compatible with the possible existence of such a complex because, as discussed before, each MCM2 is sufficient for histone binding and does not interfere with the binding of another MCM2 molecule. In addition, this asymmetrical complex proved stable at high salt concentration (Supplementary Figures S9 and S10). After histone binding by MCM2, the histone chaperone ASF1 could separate the histone H3-H4 tetramer in a second step, and stay associated with MCM2, forming the quaternary complex that we have characterized *in vitro* (Figure 4, panel 2). Such a complex was indeed shown to accumulate under replication arrest with hydroxylurea (HU) in human cells (20). Its function could be to protect histone binding surfaces from nucleosome reassembly upstream the replication fork or from unwanted interactions (Figure 4, panel 2). This mechanism raises questions concerning the dissociation of parental histone tetramers. ASF1 dissociates the tetramer in two dimers. However, parental histones H3.1 were not found to mix with newly synthesized histones during replication, arguing that tetramers do not split upon transfer through the replication fork (13). To reconcile this paradox, one has to imagine that histone dimers could transiently split, but could be maintained in close proximity by some factor of the replisome to be determined and immediately reassembled downstream of the replication fork. An ideal candidate for holding the two dimers together is the histone chaperone CAF-1. CAF-1 is itself part of the replication fork through its interaction with PCNA, it interacts with ASF1 (58) and it is well established that ASF1 transfers histones H3-H4 to CAF-1 (22,51,58) (Figure 4, panel 3). Moreover, it has been shown that CAF-1 assembles two histone H3-H4 dimers on DNA (50,54). Interestingly, histone interaction with CAF-1 requires their partial unfolding (53,59). We have shown that both ASF1 and MCM2 partially destabilize intramolecular interactions of the H3-H4 complex: MCM2 competes with the docking of the αN helix of histone H3 onto the α1 helix of histone H4 (Figure 2B) while ASF1 modifies the position of the histone H4 C-terminus that docks with helix 3 of histone H4 in the nucleosome (12). Thus, in addition to protecting histones from promiscuous interactions at the replication fork, ASF1 and MCM2 could also promote histone flexibility facilitating their management by CAF-1. Alternatively, to account for the absence of histone mixing during replication, after interaction with MCM2 in a tetrameric form, histones could be transferred directly to DNA or to CAF-1 for reassembly (Figure 4, panel 3).

The quaternary complex composed of MCM2, ASF1, H3 and H4 was also isolated from low salt human cell extracts that correspond to proteins not associated with the chromatin (14,20). Interestingly, this complex does not include MCM3–7, suggesting that MCM2 could have a role independent from its function at the replication fork. The major fraction of histones found in this complex carries the specific marks of newly synthesized histones (acetylated H4K5 and H4K12 (20,60)). The function of this soluble MCM2-H3-H4-ASF1 complex remains to be determined. Our finding that MCM2 and ASF1 cover a large histone surface of histone dimers and prevent aggregation or unwanted interactions suggest that ASF1 and MCM2 could escort new histones and participate in their delivery to CAF-1 for further assembly on DNA (Figure 4, panel 3).

In conclusion, our results highlight how MCM2 can play multiple roles in handling histones H3-H4. The structure of the ternary complex reveals that the N-terminal domain could behave as a chaperone competing with both DNA and H2B binding. This entity is covalently integrated as a component of the replication machinery probably to ensure a direct coupling between fork progression and histone management.

## ACCESSION NUMBERS

The atomic coordinates and structure factors have been deposited in the protein data bank under accession 4UUZ.

## SUPPLEMENTARY DATA

Supplementary Data are available at NAR Online.

SUPPLEMENTARY DATA
