# Supplementary material for: Structural insight into how the human helicase subunit MCM2 may act as a histone chaperone together with ASF1 at the replication fork
Source: Nucleic Acids Res. 2015 Jan 23;43(3):1905–17. doi: 10.1093/nar/gkv021 (PMC4330383; doi:10.1093/nar/gkv021)
Supplement: SUPPLEMENTARY DATA [file supp_43_3_1905__index.html]

Structural insight into how the human helicase subunit MCM2 may act as a histone chaperone together with ASF1 at the replication fork — SUPPLEMENTARY DATA 

# Structural insight into how the human helicase subunit MCM2 may act as a histone chaperone together with ASF1 at the replication fork

## SUPPLEMENTARY DATA

**Files in this Data Supplement:**

- SUPPLEMENTARY DATA
