# Supplementary material for: Structural insight into how the human helicase subunit MCM2 may act as a histone chaperone together with ASF1 at the replication fork
Source: Nucleic Acids Res. 2015 Jan 23;43(3):1905–17. doi: 10.1093/nar/gkv021 (PMC4330383; doi:10.1093/nar/gkv021)
Supplement: SUPPLEMENTARY DATA [file supp_gkv021_Richet_Sup_figures_review2.pdf]

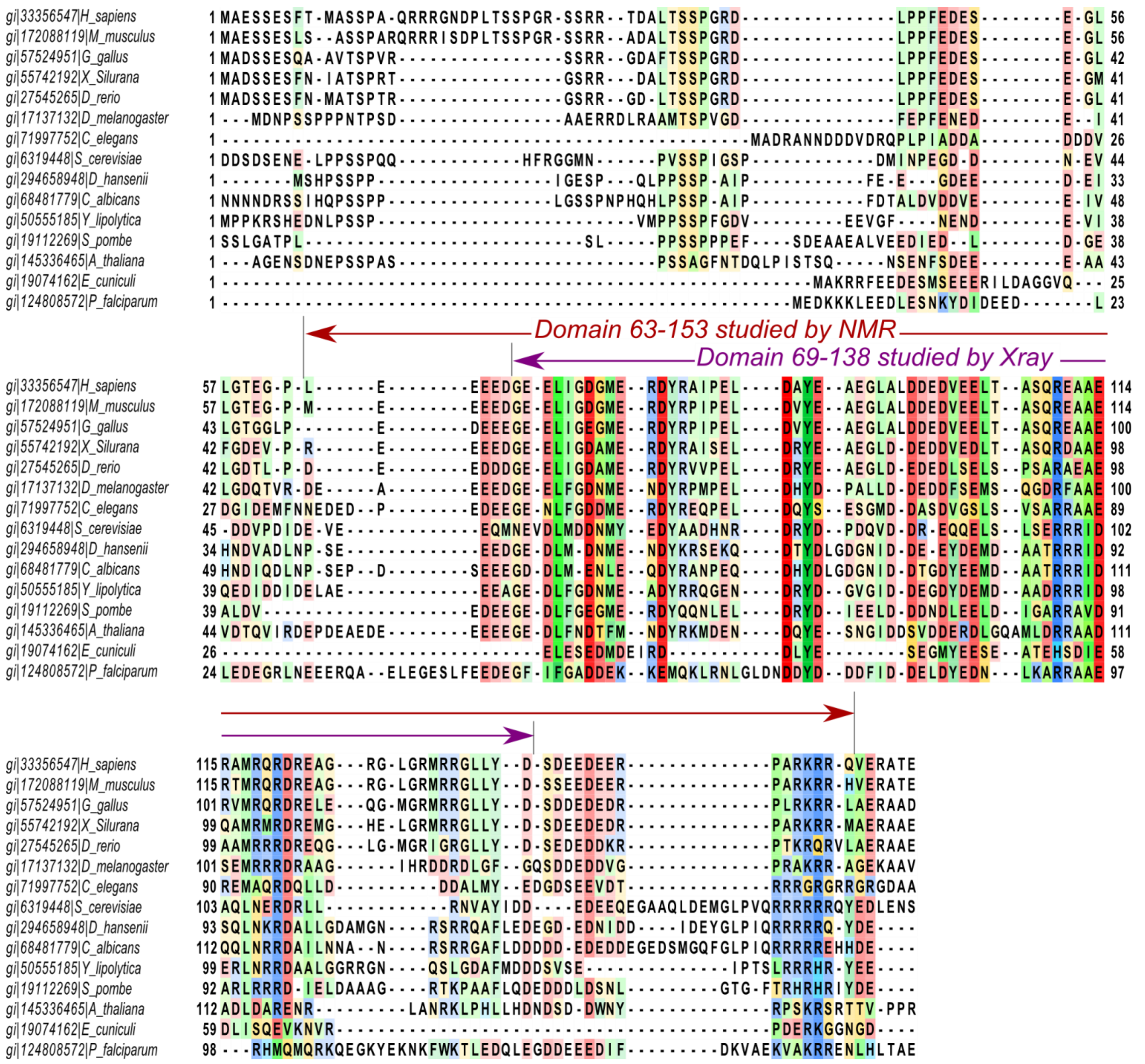

Supplementary Figure S1 : Multiple sequence alignment of MCM2 orthologs

Multiple sequence alignment of MCM2 orthologs in 15 eukaryotic species focused on the 1-160 N-terminal region and computed using Mafft E-INS-i algorithm (1). Columns are shaded with respect to their conservation degree coloring residues with respect to their physic-chemical properties : acidic (red), basic (blue), aliphatic (light green), aromatic (dark green) and uncharged polar (yellow) (represented using Jalview (2))

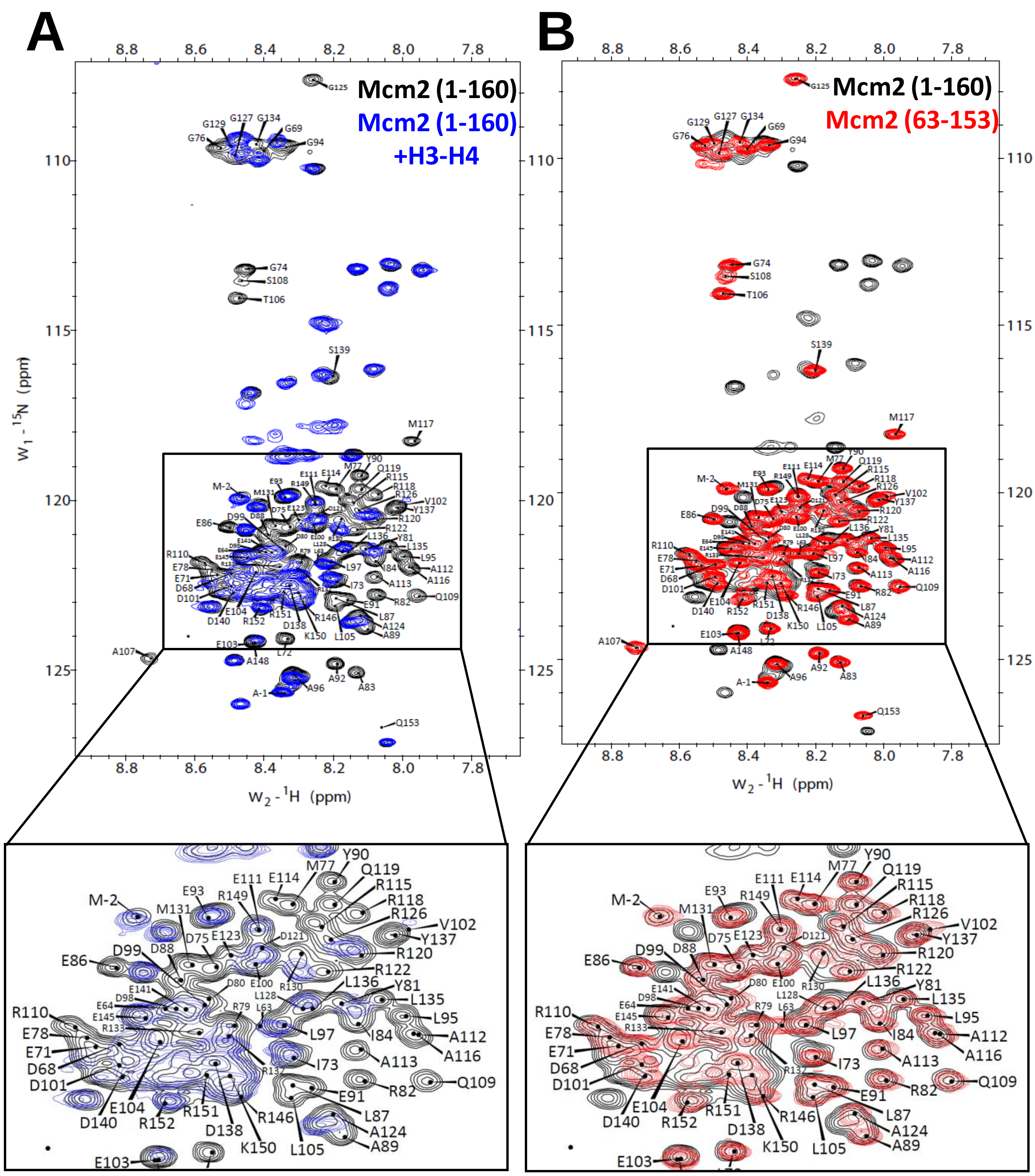

Supplementary Figure S2 : NMR characterization of the MCM2 histone binding domain

(A) Overlay of the ^{1}H-^{15}N HSQC spectra of MCM2(1-160) free (black) and bound to an excess of H3–H4 tetramer (blue). Spectra were recorded at 20°C, in a Tris 50mM buffer pH 8, 1.5 M NaCl. Signals disappearing in the bound form report for residues of MCM2 involved in the interaction with histones,

(B) Overlay of the ^{1}H-^{15}N HSQC spectra of free MCM2(1-160) (black) and MCM2(63-153) (red). Black signals only present in the longer fragment were not affected upon addition of histones in panel B showing that the histone binding region lies within the shorter fragment,

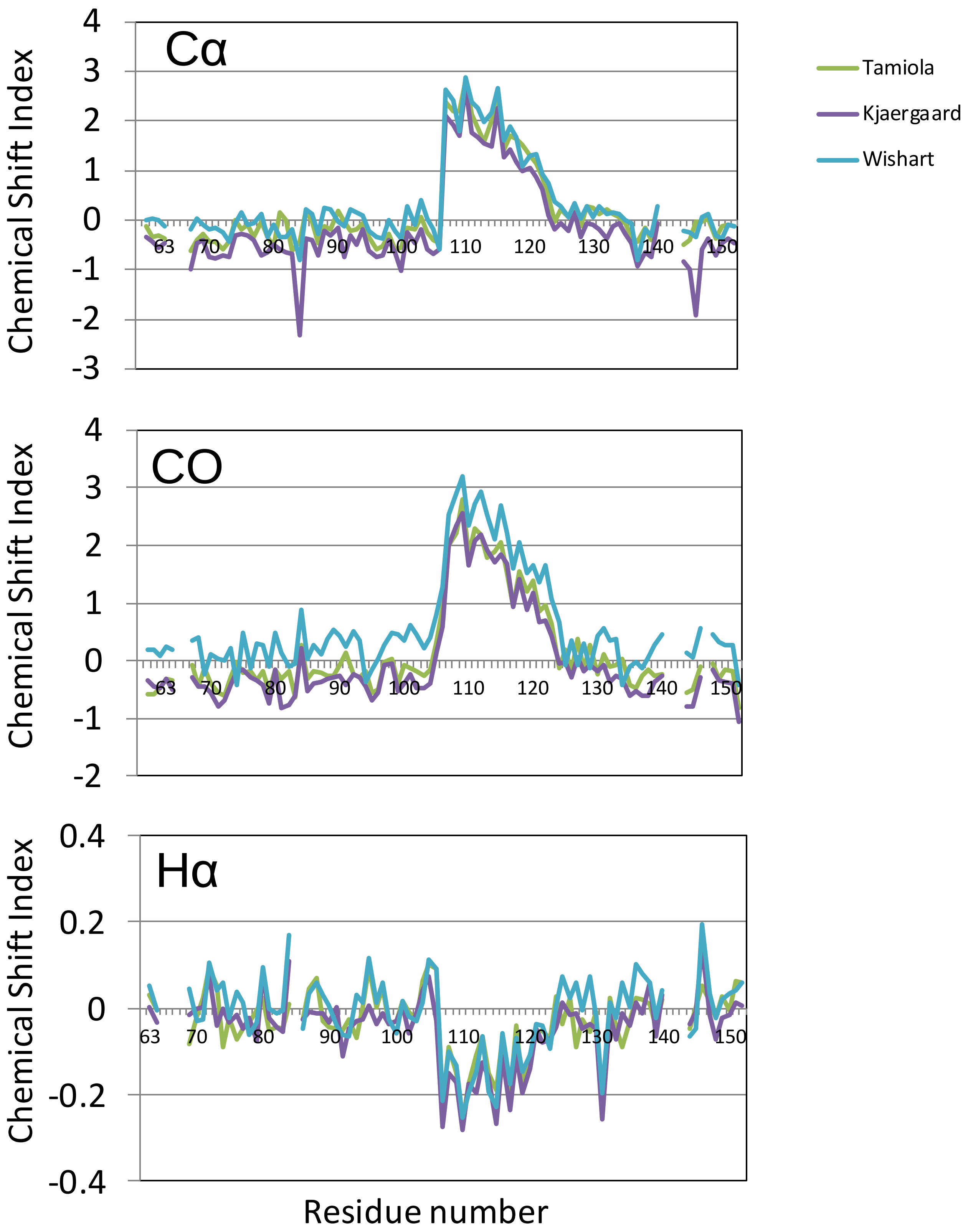

Supplementary Figure S3 : Chemical shift indexes of free MCM2(63-153)

Chemical shift indexes (CSI) along the sequence of free MCM2(63-153) using resonances of three reporter atoms ^{13}Cα , CO, Hα and plotted with three reference sets for the unfolded state Tamiola (green), Kjaergaard (purple), Wishart (blue) (see Methods for details)

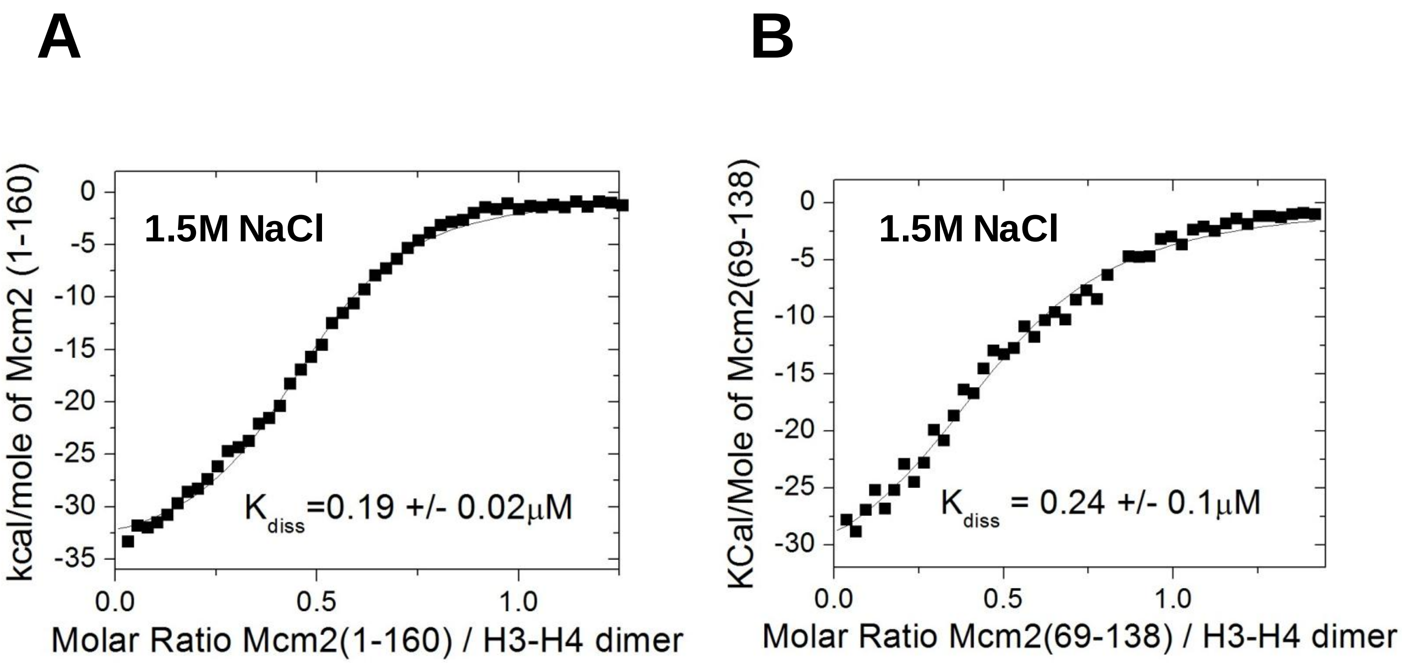

Supplementary Figure S4 : Affinity measurement of two MCM2 N-terminal segments for histones H3-H4 at 1.5M NaCl

(A) Dissociation constant (*Kdiss*) of MCM2(1-160) in complex with histones H3-H4 as determined by isothermal calorimetry in 1.5 M NaCl, Tris 50mM pH 8, at 20°C,

(B) Dissociation constant (*Kdiss*) of MCM2(69-138) in complex with histones H3-H4 as determined by isothermal calorimetry in 1.5 M NaCl, Tris 50mM pH 8, at 20°C.

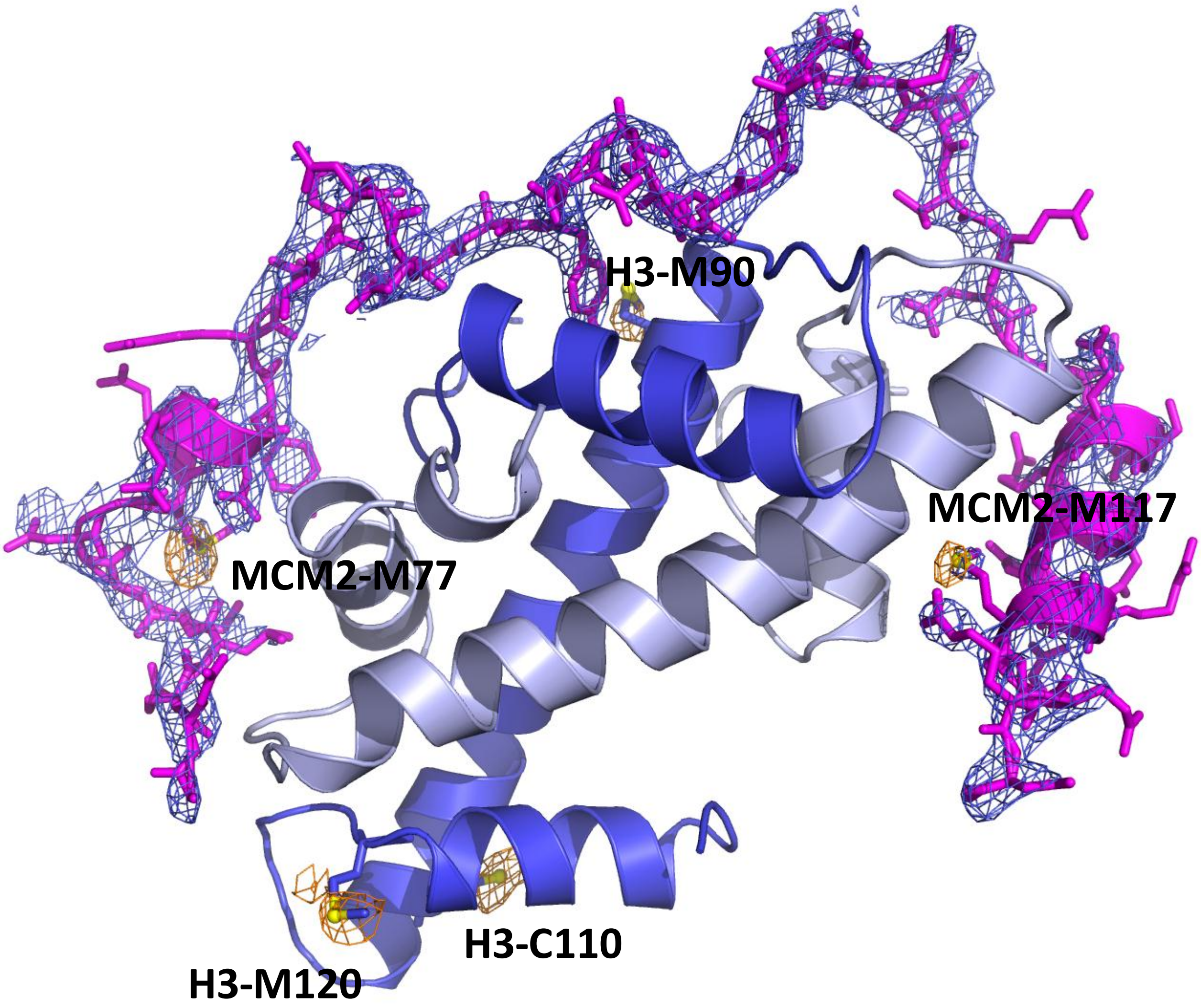

Supplementary Figure S5 : Structure of the ternary complex containing two MCM2 histone-binding domains (69-121) bound to a tetramer of histones H3 and H4

Ribbon representation of the H3-H4-MCM2 ternary complex. H3 and H4 are colored in dark blue, and light blue while the MCM2 chain is represented in magenta sticks. Weighted electron-density maps around MCM2 is colored in blue and contoured at 1.0 σ. Anomalous difference map calculated by AnoDe (3) at 4.2-Å is contoured at 3 σ and rendered in orange. Sulfur atoms are highlighted in yellow. Anomalous peaks (in σ) for sulfur atoms are at 5.3 and 3.9 for M77 and M117 respectively in the MCM2 chain and at 3.9, 4.3 and 4.4 for M90, C110 and M120 respectively in the H3 chain.

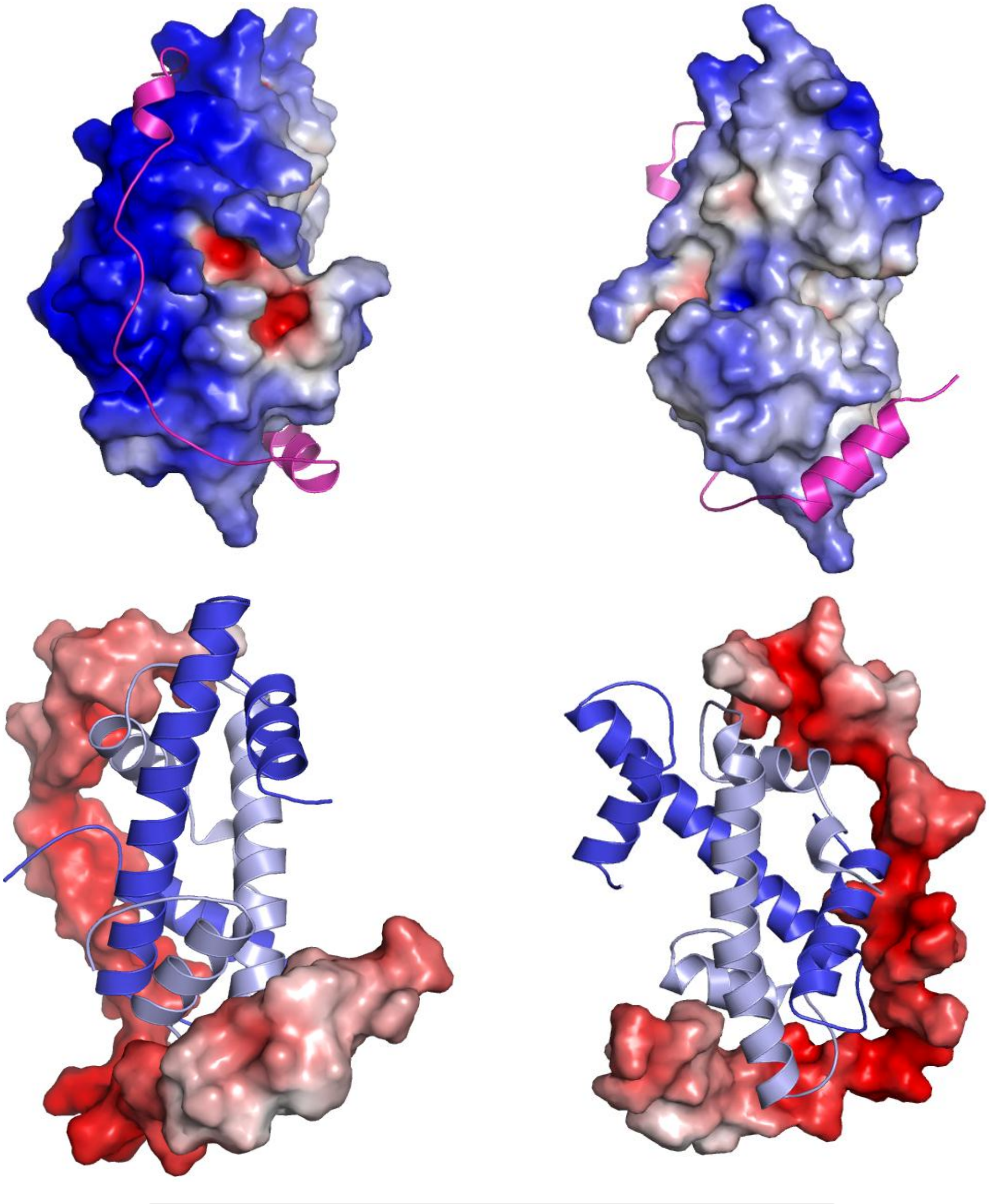

Supplementary Figure S6 : Electrostatic potentials in the MCM2–H3–H4 complex

Electrostatic potential mapped at the solvent accessible surfaces of the histones H3–H4 heterodimer (top) and of MCM2(69-121) (bottom) in the context of the ternary MCM2–H3–H4 complex (from red (negative charge) to blue (positive charge) calculated using APBS method (4). Cartoon representations of MCM2 (magenta), histones H3 (dark blue) and H4 (light blue) delineate the binding interface

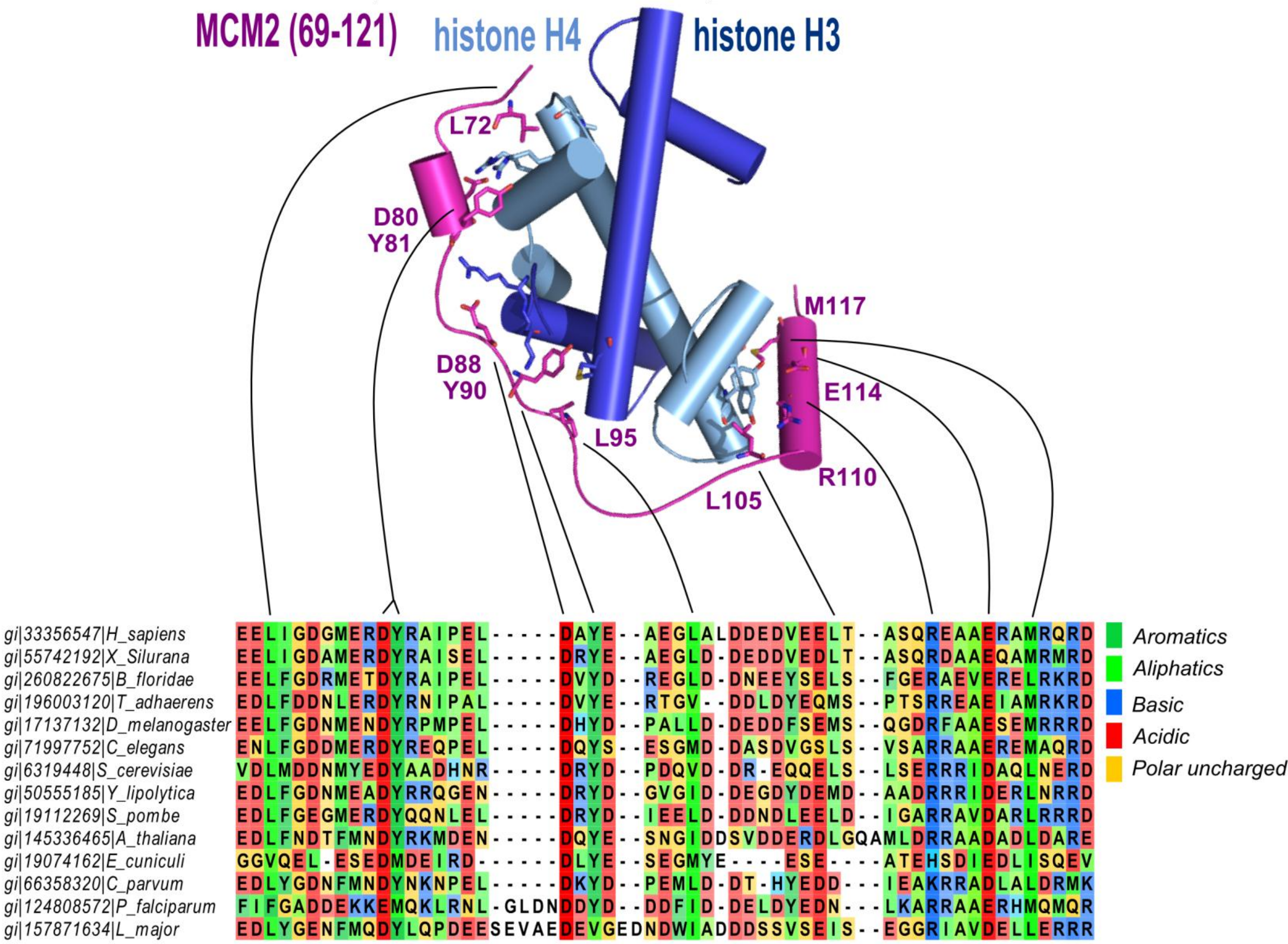

Supplementary Figure S7 : Conservation of MCM2 residues involved in histone binding.

Structure of the ternary MCM2(magenta)–H3(dark blue)–H4(light blue) complex highlighting the side-chains of MCM2 making extensive contacts with either histones H3 or H4. The conservation of the corresponding positions in the multiple sequence alignment of MCM2 orthologs in 14 representative eukaryotic species is depicted below. Species were selected to present the most diverged set of sequences highlighting the contrast between invariant and variable positions. Alignment was carefully built from a larger set of 209 sequences by assembling in a hierarchical manner the sequence profiles generated for the various clades (animals, fungi, plants, parasites) using the mafft-profile algorithm (1). Columns are shaded with respect to their conservation degree coloring residues with respect to their physic-chemical properties : acidic (red), basic (blue), aliphatic (light green), aromatic (dark green) and uncharged polar (yellow) (represented using Jalview (2).

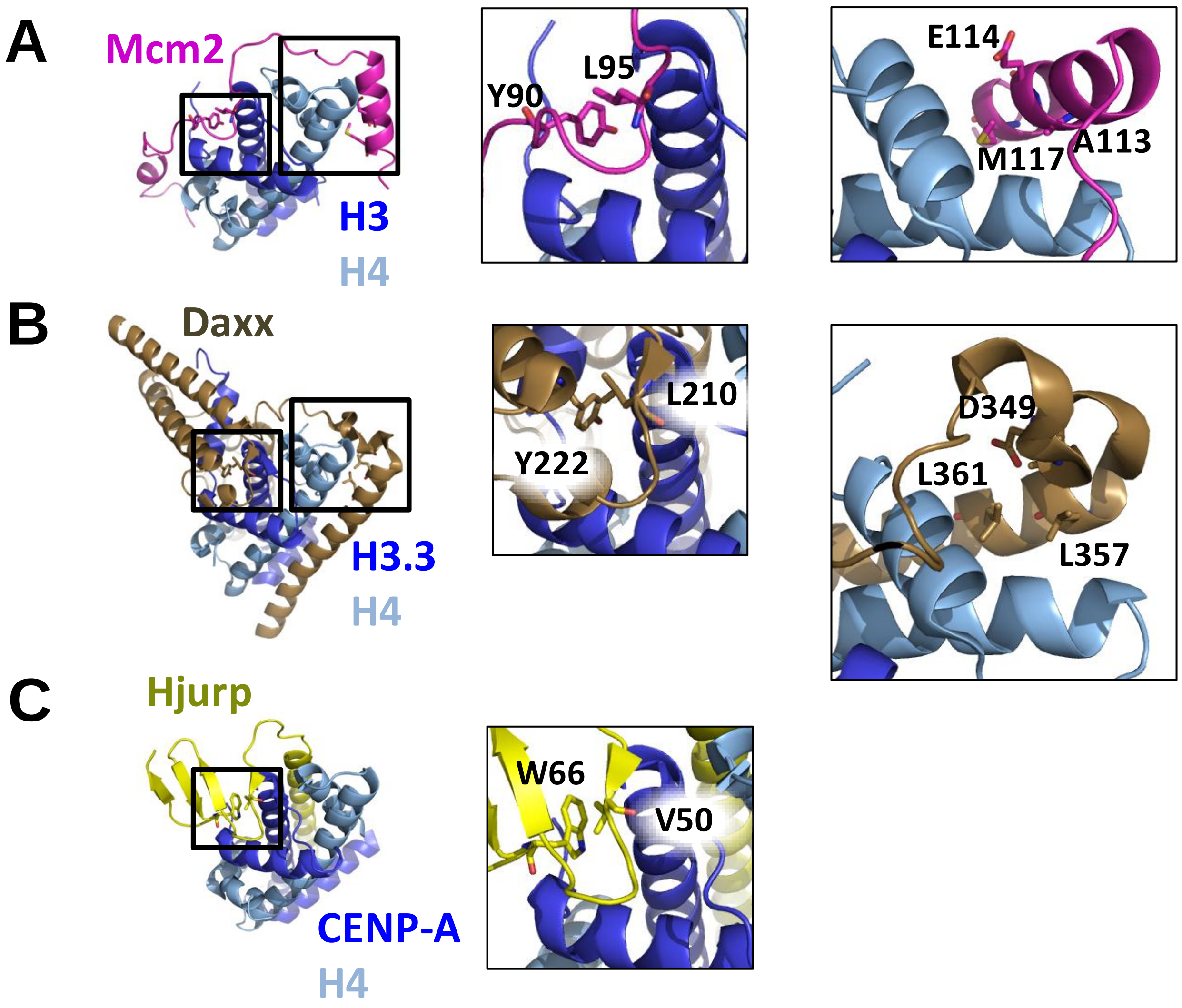

Supplementary Figure S8 : H3-H4 binding mode of MCM2 compared with that of other histone chaperones

Close-up views comparing how MCM2 (magenta) (A) , DAXX (brown) (B) and HJURP (yellow) (C) interact with different variants of histones H3-H4 in equivalent sites (i) at the interface of helices α1 and α2 of histone H3 (middle panels) and (ii) at the interface of helices α2 and α3 of histone H4 (right panels) (PDB codes 4HGA and 3R45 for DAXX–H3.3–H4 and HJURP–CENP-A–H4 complexes, respectively).

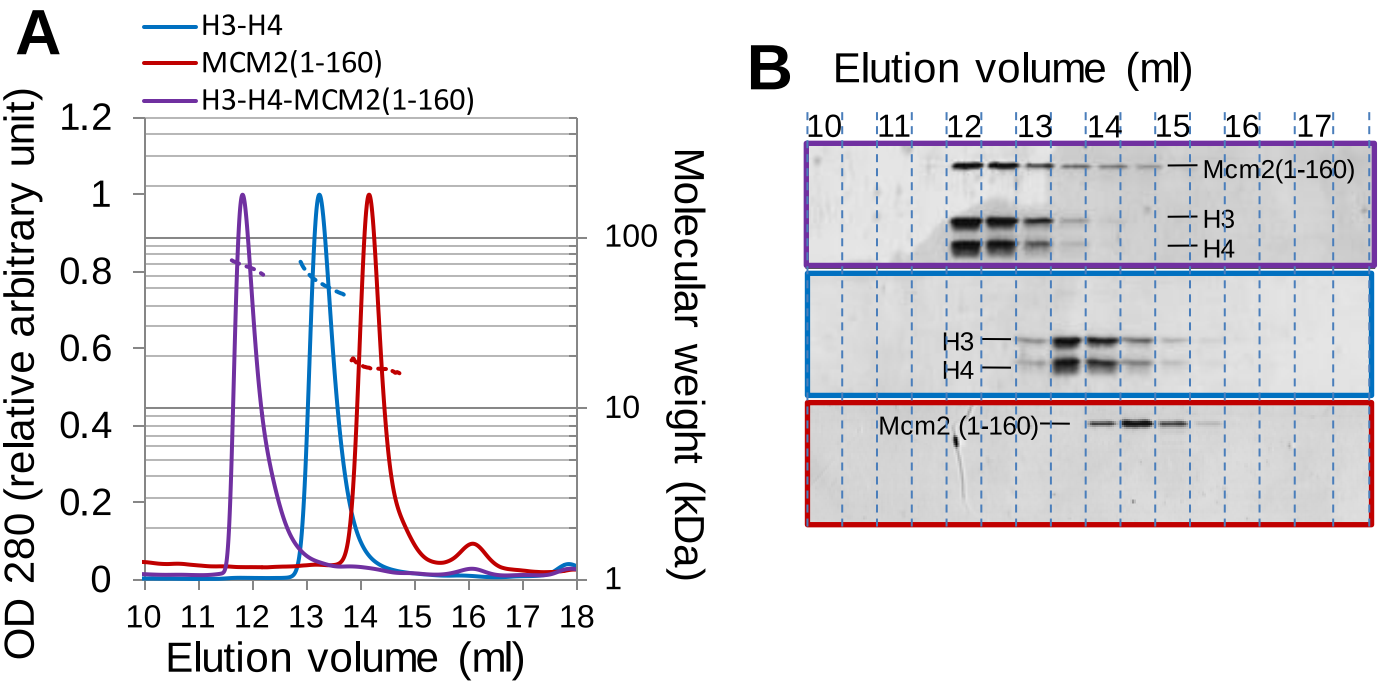

Supplementary Figure S9 : Sec-MALS analysis of MCM2 (1-160)-H3-H4 at 1.5M NaCl

(A) Sec-MALS analysis at 20°C in a Tris 50mM buffer pH 8, 1.5 M NaCl, of H3-H4 (in blue), MCM2(1-160) in red and H3-H4-MCM2(1-160) in purple. Relative optical density at 280nm was plotted in arbitrary units in continuous lines as a function of the elution volume. The calculated molecular mass is reported as dashed line in the corresponding color using the secondary scale on the right.

(B) SDS-PAGE analysis of Sec-MALS fractions for the different samples collected in A with a color code as in A

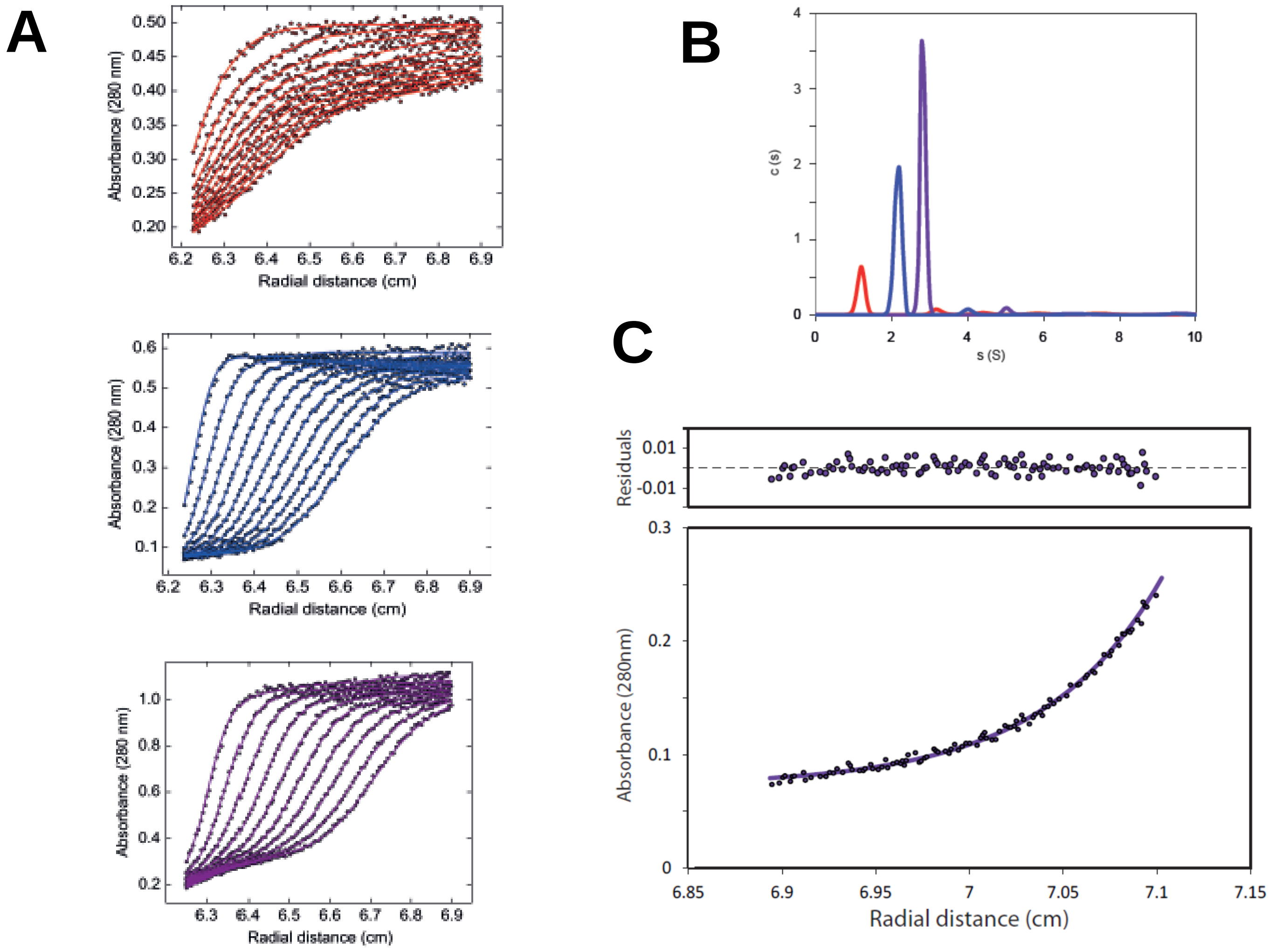

Supplementary Figure S10 : AUC characterization analysis of MCM2 (1-160)-H3-H4 at 1.5M NaCl

(A) Sedimentation velocity of H3-H4 (28 µM, blue curves), MCM2(1-160) (100µM, red curves), and H3-H4-MCM2 (100 µM and 100 µM, respectively, purple curves) in 50 mM Tris, 1.5M NaCl, pH 8.0. The positions of the moving boundaries shown were recorded at 5-min intervals. The solid lines are best fits of the experimental data (◦)

(B) sedimentation coefficient distribution, c(s) for H3-H4 (blue line), MCM2(1-160) (red line) and H3-H4-MCM2(1-160) (purple line)

(C) Analytical ultracentrifugation on the H3-H4 complex. Sedimentation equilibrium profiles obtained for purified H3H4 complex. Lower panel, experimental data were obtained for proteins at absorbance at 280nm of 0.2 and rotor speed of 21768g (solid line) in a Beckman Coulter XL-A analytical ultracentrifuge. A global fit to different concentrations of H3-H4 at three different speeds assuming a one species analysis is shown. Upper Panel, the corresponding distribution of the residual is shown in the plot.

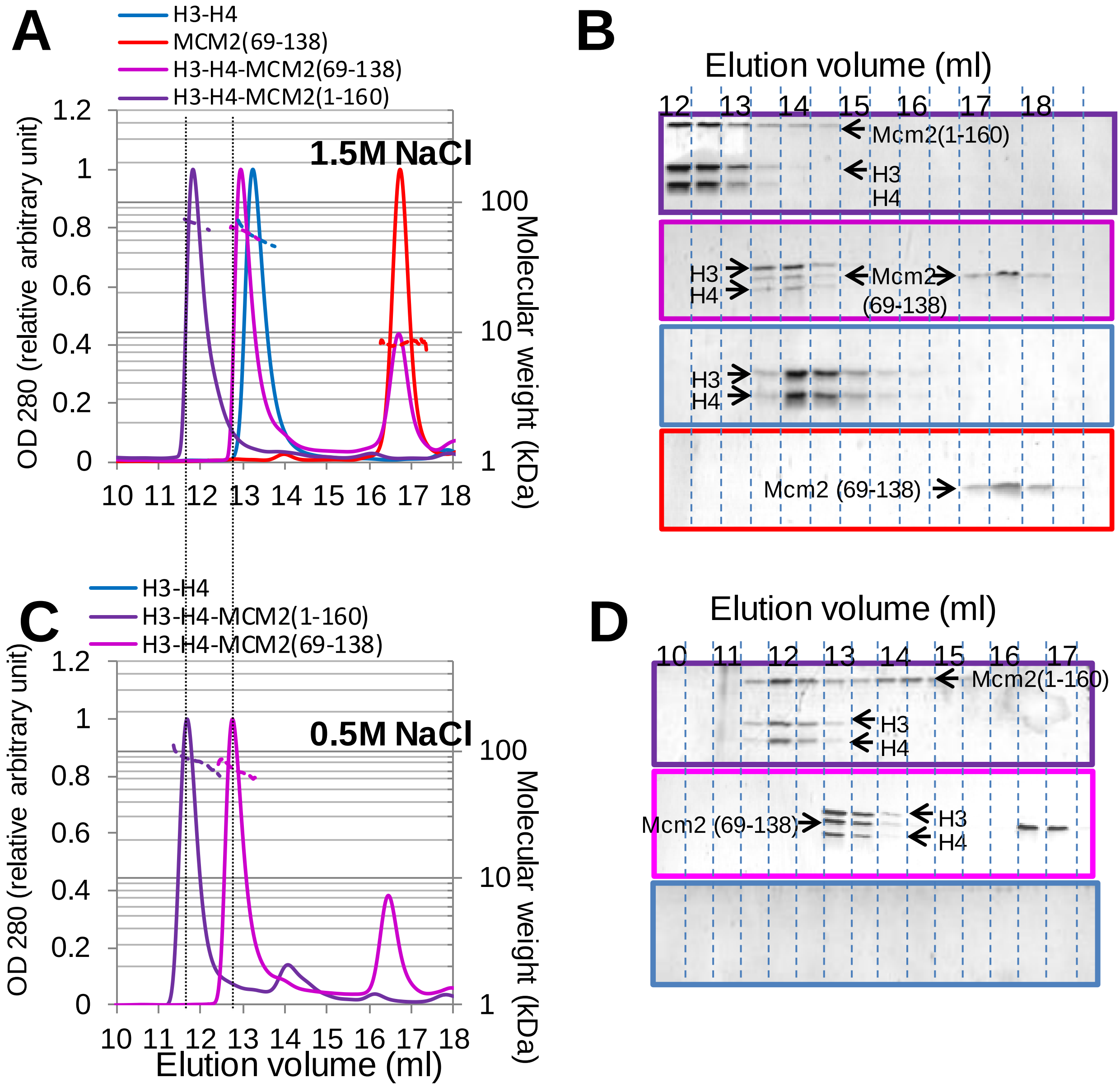

Supplementary Figure S11 : Sec-MALS analysis of two MCM2 N-terminal segments and Histones H3-H4 at different salt concentrations

(A) Sec-MALS analysis at 20°C in a Tris 50mM buffer pH 8, 1.5 M NaCl, of H3-H4 (in blue), MCM2(69-138) in red H3-H4-MCM2(69-138) in magenta and H3-H4-MCM2(1-160) in purple. Units are as in A,

(B) SDS-PAGE analysis of Sec-MALS fractions for the samples collected in F with a color code as in A

(C) Sec-MALS analysis at 20°C in a Tris 50mM buffer pH 8, 0.5 M NaCl, of H3-H4-MCM2(69-138) in magenta and H3-H4-MCM2(1-160) in purple. Units are as in A. A vertical dashed line is added to highlight the pic displacement compared to the measures in 1.5M NaCl. Peaks are shifted to lower values of elution volume and present higher molecular masses consistent with the modification of the complex stoichiometry upon ionic strength variations (see Table 1) the chromatogram for free H3-H4 is not shown because it was flatt. All the sample precipitated before injection in the column

(D) SDS-PAGE analysis of Sec-MALS fractions for the samples collected in H with a color code as in C

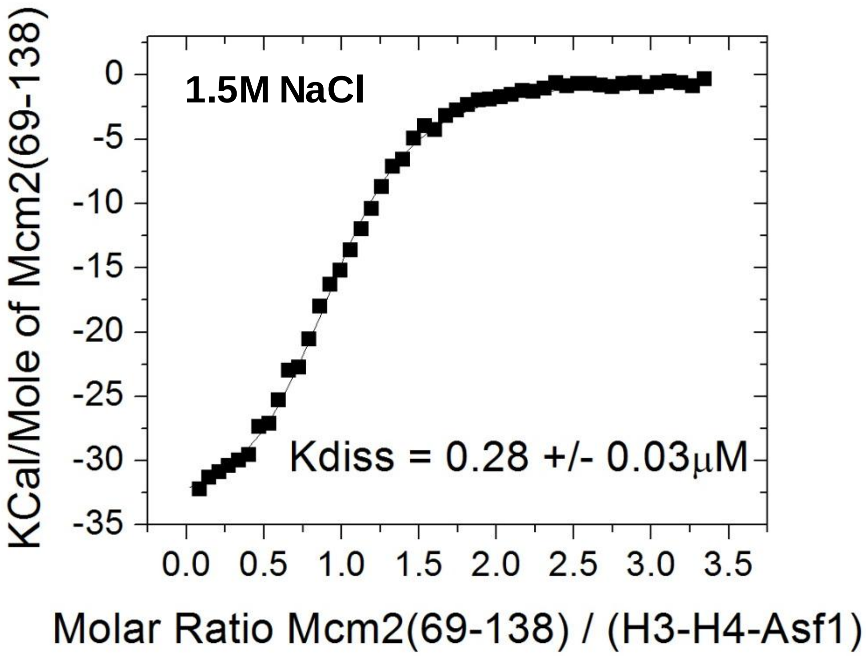

Supplementary Figure S12 : Affinity measurement of MCM2(69-138) for histones ASF1-H3-H4 at 1.5M NaCl

Dissociation constant (Kdiss) of MCM2(69-138) in complex with ASF1A(1-156)-H3-H4 as determined by isothermal calorimetry in 1.5 M NaCl, Tris 50mM pH 8, at 20°C,

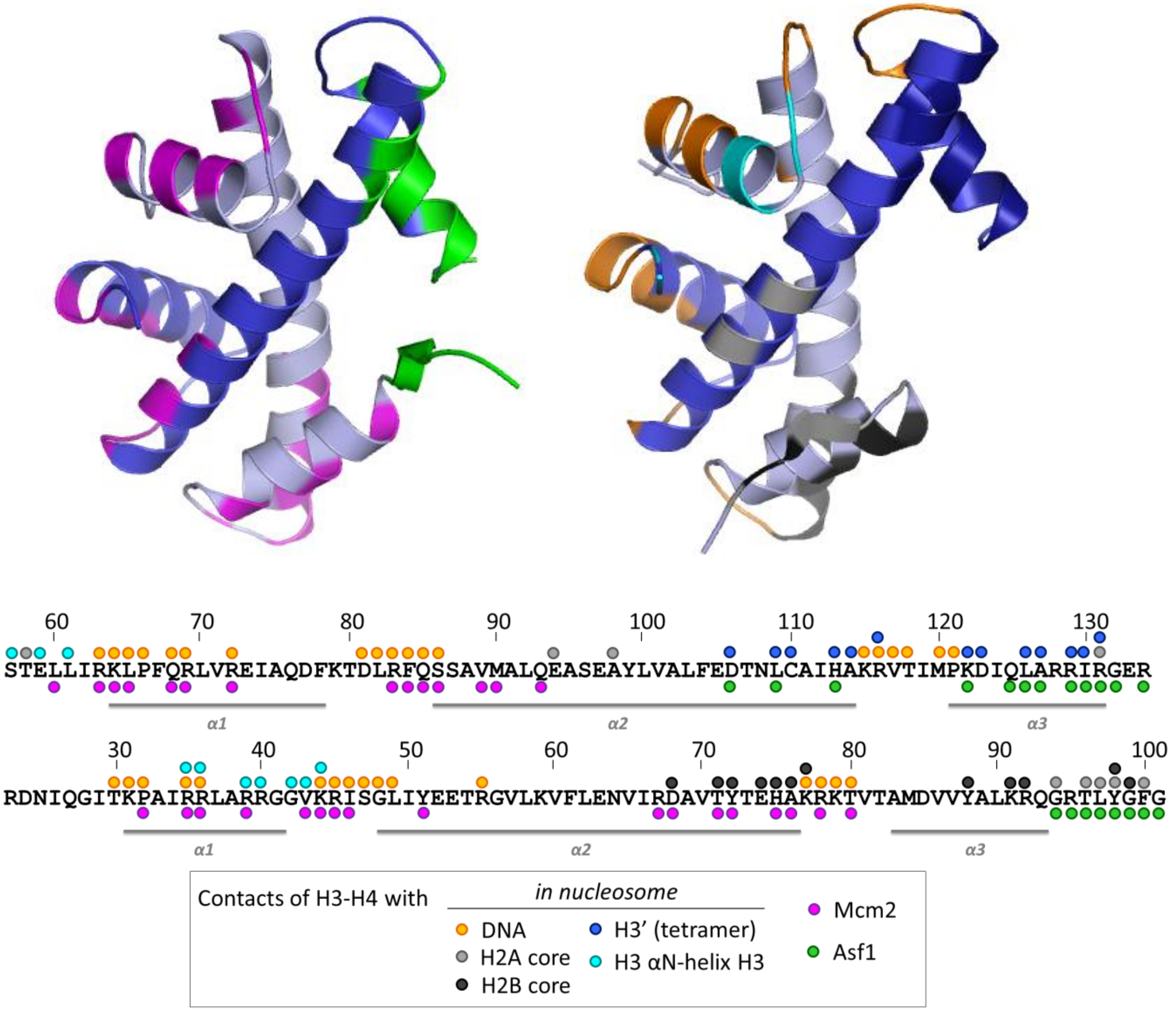

Supplementary Figure S13 : Mapping of MCM2 residues in contact with different partners in the nucleosome and in the ASF1-H3-H4-MCM2 complex

Altogether, ASF1(1-516) and MCM2(69-121) contact histones H3 and H4 on all their surface zones which are involved in intermolecular interactions within the entire nucleosome. The only exception lies in the K_{115}-P_{121} stretch of histone H3 contacting DNA and termed the diad region. Residues of histones H3 and H4 are labelled by colored circles with respect to the nature of the macromolecule they are bound to. Contacts of histones H3 and H4 with MCM2(69-121) (this work), ASF1A(1-156) (PDB 2IO5), histones H2A, H2B, H3', H3 and DNA (PDB 1KX5) are shown in magenta, green, light gray, dark gray, blue, cyan and yellow, respectively.

Supplementary Table S1 : Affinity measurements by ITC

Protein in the cell	Protein in the syringe[£]	Salt concentration [$]	Kd (μM)	ΔG ($kCal.M^{-1}$)	N*	ΔH ($kCal.M^{-1}$)	-TΔS ($kCal.M^{-1}$)
H3-H4	$MCM2_L$	NaCl 1.5M	0.19(±0.02)	-8.50(±0.03)	0.49(±0.03)	-34.8(±0.5)	26.3(±0.5)
H3-H4	$MCM2_S$	NaCl 1.5M	0.24(±0.1)	-8.4(±0.5)	0.50(±0.01)	-35.1(±1.2)	26.7(±0.2)
H3-H4-ASF1	$MCM2_S$	NaCl 0.5M	0.024(±0.002)	-9.67(±0.04)	1.04(±0.02)	-45.5(±0.2)	45.5(±2.4)
H3-H4-ASF1	$MCM2_S$	NaCl 1.5M	0.28(±0.03)	-8.3(±0.06)	0.97(±0.06)	-35.4(±0.9)	27.1(±0.9)

[$] Buffer used for all experiments, Tris 50mM, pH8

[£] $MCM2_L$ states for MCM2(1-160), $MCM2_S$ states for MCM2(69-153), ASF1 states for ASF1A(1-156)

[*] binding stoichiometry

Supplementary Table S2 : Data collection and refinement statistics

	H3H4-MCM2
**Data collection**	
Space group	*R32*
Cell dimensions	
$a = b, c$ (Å)	140.1, 66.0
$\alpha = \beta, \gamma$ (°)	90.0, 120.0
Resolution (Å)	60.0-2.9 (2.97-2.9) *
R_{merge}	7.5 (205.2)
$I / \sigma I$	19.7 (1.2)
Completeness (%)	99.9 (98.8)
Redundancy	10.8 (10.5)
**Refinement**	
Resolution (Å)	2.9
No. reflections	5602
R_{work} / R_{free} (%)	19.72/ 23.03
No. atoms	
Protein	1682
Ligand/ion	0
Water	0
B-factors	
Protein	131.9
Ligand/ion	
Water	
R.m.s. deviations	
Bond lengths (Å)	0.010
Bond angles (°)	1.15

*Values in parentheses are for highest-resolution shell.

Supplementary bibliography

1. Katoh, K. and Standley, D.M. (2013) MAFFT multiple sequence alignment software version 7: improvements in performance and usability. *Mol Biol Evol*, **30**, 772-780.
2. Waterhouse, A.M., Procter, J.B., Martin, D.M., Clamp, M. and Barton, G.J. (2009) Jalview Version 2--a multiple sequence alignment editor and analysis workbench. *Bioinformatics*, **25**, 1189-1191.
3. Thorn, A. and Sheldrick, G.M. (2011) ANODE: anomalous and heavy-atom density calculation. *J Appl Crystallogr*, **44**, 1285-1287.
4. Baker, N.A., Sept, D., Joseph, S., Holst, M.J. and McCammon, J.A. (2001) Electrostatics of nanosystems: application to microtubules and the ribosome. *Proc Natl Acad Sci U S A*, **98**, 10037-10041.
